# Systematic review and network meta-analysis of the efficacy of existing treatments for patients with recurrent glioblastoma

**DOI:** 10.1093/noajnl/vdab052

**Published:** 2021-04-09

**Authors:** Anna Schritz, Nassera Aouali, Aurélie Fischer, Coralie Dessenne, Roisin Adams, Guy Berchem, Laetitia Huiart, Susanne Schmitz

**Affiliations:** 1 Competence Center for Methodology and Statistics, Department of Population Health, Luxembourg Institute of Health, Strassen, Luxembourg; 2 Clinical and Epidemiological Investigation Center, Department of Population Health, Luxembourg Institute of Health, Strassen, Luxembourg; 3 Department of Hemato-Oncology, Centre Hospitalier de Luxembourg, Luxembourg, Luxembourg; 4 Luxembourg Institute of Health, Strassen, Luxembourg; 5 Department of Population Health, Luxembourg Institute of Health, Strassen, Luxembourg; 6 National Centre for Pharmacoeconomics, Dublin, Ireland

**Keywords:** network meta-analysis, overall survival, progression-free survival, recurrent glioblastoma, systematic review

## Abstract

**Background:**

Despite advances in the treatment of cancers over the last years, treatment options for patients with recurrent glioblastoma (rGBM) remain limited with poor outcomes. Many regimens have been investigated in clinical trials; however, there is a lack of knowledge on comparative effectiveness. The aim of this systematic review is to provide an overview of existing treatment strategies and to estimate the relative efficacy of these regimens in terms of progression-free survival (PFS) and overall survival (OS).

**Methods:**

We conducted a systematic review to identify randomized controlled trials (RCTs) investigating any treatment regimen in adult patients suffering from rGBM. Connected studies reporting at least one of our primary outcomes were included in a Bayesian network meta-analysis (NMA) estimating relative treatment effects.

**Results:**

Forty RCTs fulfilled our inclusion criteria evaluating the efficacy of 38 drugs as mono- or combination therapy. Median OS ranged from 2.9 to 18.3 months; median PFS ranged from 0.7 to 6 months. We performed an NMA including 24 treatments that were connected within a large evidence network. Our NMA indicated improvement in PFS with most bevacizumab (BV)-based regimens compared to other regimens. We did not find any differences in OS between treatments.

**Conclusion:**

This systematic review provides a comprehensive overview of existing treatment options for rGBM. The NMA provides relative effects for many of these treatment regimens, which have not been directly compared in RCTs. Overall, outcomes for patients with rGBM remain poor across all treatment options, highlighting the need for innovative treatment options.

**Key Points**

- Treatment options for recurrent glioblastoma show negligible improvement in overall survival (OS).
- Differences were marginally more evident for progression-free survival (PFS) versus OS.
- Bevacizumab mono- and combination therapies show improved PFS.

**Importance of the Study**

Given the large number of possible treatment options for recurrent glioblastoma, it is important to understand the comparative efficacy between these treatments. Undertaking evidence synthesis of the available trials allows this to be done in a systematic way. This literature review and Bayesian network-meta analysis investigates the current evidence of available treatment options and their efficacy to treat patients with recurrent glioblastoma. We systematically searched the literature for any randomized controlled trial investigating any treatment option for adults with recurrent glioblastoma. Given the scarcity of direct comparative evidence, network meta-analysis provides a means of estimating comparative treatment effects based on direct as well as indirect evidence. Our analysis is the largest network meta-analysis conducted in this patient population to date, estimating relative treatment effects of 24 distinct treatment regimens. Overall, our analysis highlights the need for innovative treatment options for recurrent glioblastoma, as outcomes for patients remain poor across treatment options.

Among primary brain tumors, gliomas represent around 80% of brain cancers. Glioblastoma (GBM) (grade 4 glioma according to the World Health organization (WHO) classification^1^) is the most common and aggressive brain tumor in adults. With an incidence of GBM of 3.2 in 100,000 people,^2^ the cancer affects all ages of the population. Despite an armamentarium of therapies developed, relapse of GBM is inevitable for almost all patients; median survival is around 1 year after recurrence.^3^

The diagnosis of recurrence in GBM is still challenging. The resistance to treatment of tumor cells, the heterogeneity and evolution of subclonal populations of cancer cells constituting the tumor as well as the genetic features of tumor cells seem to be the pillar of progression and failure of therapy.^4^

Treatment options upon progression are limited, with no standard of care clearly defined. Recurrent glioblastoma (rGBM) patients are recommended to enroll in clinical studies where possible.^5^ Treatment choice is guided by several factors including performance status,^6^ tumor size, and location.^7–9^

Only a small percentage of patients are eligible for re-operation.^10,11^ The benefit of salvage surgery on survival depends on performance status,^10^ tumor location, initial resection status, and age at relapse of GBM patients.^11,12^ With the availability of improved imaging technology, re-irradiation is another option that is used in the treatment of rGBM. However, stringent criteria are applied including tumor size, tumor resection size, age, prior therapy, and the time between irradiation and re-irradiation.^9^ The third option is systemic treatment. Drugs mostly used are alkylating or anti-angiogenic agents alone or in combination with other molecules. One of the anti-angiogenic drugs, bevacizumab (BV) is the only drug licensed for rGBM by the Food and Drug Administration (FDA) in the United States (since 2009).^13^ BV has not been approved by the European Medicines Agency (EMA) owing to the lack of sufficient and convincing data.^14^ The high level of vascularization and expression of vascular endothelial growth factor (VEGF) in rGBM supports the use of BV. Alkylating agents, such as nitrosoureas (carmustine [BCNU] or lomustine [CCNU]) are used for their lipophilic properties; they were the first drugs used in the treatment of rGBM^3^ before the FDA approved BV. Recognized as a reference drug in the treatment of newly diagnosed patients,^15^ the use of temozolomide (TMZ) appears challenging in the treatment of rGBM patients with MGMT (O^6-^methylguanine-DNA-methyl-transferase) promoter methylated tumors.

Meta-analysis allows analyzing data from multiple trials comparing the same two interventions simultaneously producing an overall effect of relative efficacy.^16,17^ Network Meta-analysis (NMA) is a natural extension of the methodology to allow for the estimation of relative efficacy within a network of multiple interventions. The methodology makes use of direct and indirect evidence and is useful where multiple treatment options exist, which have not been directly compared or where head-to-head evidence is insufficient.^18–20^ Several meta-analyses on the topic exist; however, these evaluated the efficiency of only one treatment strategy compared to another,^8,21–24^ as well as few NMA analyses including a small subset of treatment options.^25–27^

Until now, no NMA aiming to compare a large number of available therapies for rGBM exists. Thus, we have conducted an extensive systematic review of the literature and fitted a large NMA incorporating all connected treatment regimens investigated to treat rGBM in an RCT setting. The objective of this analysis is to (i) provide an overview of treatment regimens evaluated for use in rGBM in an RCT setting and their associated efficacy and (ii) estimate the relative efficacy between treatment regimens using a Bayesian NMA. Outcomes considered for this analysis are progression-free survival (PFS) and overall survival (OS).

## Material and Methods

### Search Strategy and Selection Criteria

This systematic review was performed according to the Preferred Reporting Items for Systematic Reviews and Meta-analysis (PRISMA) criteria.^28^ The review is registered with PROSPERO (CRD42019142695).

A systematic search of the published literature was conducted from inception to July 2019; search results were updated in March 2020 to identify eligible studies using EMBASE, MEDLINE (via PubMed) and CENTRAL (via Cochrane library) databases. The search was complemented by a search of the clinical trial register (clinicaltrials.gov). Systematic reviews on the topic were hand-searched for additional trials.

Inclusion criteria for the systematic review were RCTs of adult patients (≥18 years) with rGBM investigating any intervention compared to either placebo or an active comparator. Only trials reporting OS, PFS, or tumor response were included. The full search strategy can be accessed as Supplementary Material 1.

Two independent reviewers (A.S./A.F. or N.A./S.S.) screened each article. Inconsistencies during title/abstract screening and full-text screening were resolved in discussion between both reviewers or by a third reviewer in cases where no agreement was found.

A data extraction form was developed using Microsoft Word to create a form with fillable fields with Adobe Acrobat Pro. The final format was agreed upon following piloting a first version using 8 articles. We extracted general information about the study record, questions about eligibility of the study in the systematic review (Population, Intervention, Comparison, Outcomes and Study (PICOS)), information and setting of the study population, results of the outcomes of interests and information about applicability of the study to the review. The full form can be accessed as Supplementary Material 2. Two independent reviewers also performed data extraction. Where no confidence intervals or variance measures of median OS or median PFS were provided, available Kaplan–Meier plots and event tables were digitized to recreate the underlying numerical data using WebPlotDigitizer.^29^ A suitable R function was applied to recreate independent patient data to calculate Kaplan–Meier estimates.^2^

### Clinical Endpoints

For the NMA median OS and median PFS were chosen, as they were the most widely reported outcomes. Other endpoints, that were extracted but not further analyzed due to low number of reporting, were 6-month PFS, 12-month PFS, 6-month OS, 12-month OS, and tumor response rates. Tumor response rates were assessed as overall response (complete response + partial response), complete response, partial response, and stable disease.

### Statistical Analysis

Study characteristics were described using means or frequencies. We performed fixed-effects Bayesian NMA including 24 treatments that were connected within a large evidence network for OS and 23 for PFS. An NMA analyses an entire network of treatments estimating relative treatment effects between all pairwise comparisons in the network utilizing direct and indirect evidence. The inclusion of a large evidence base fits naturally in a Bayesian framework, which supports conclusions based on all available information.^18^ Based on median PFS data and patient numbers, the model estimated the relative efficacy for each pairwise comparison, measured as hazard ratios (HRs) assuming an exponential survival model, as has been done previously.^30^ Noninformative priors, following a normal distribution with a mean equal to 0 and precision set to 0.01, were used. A fixed effects model was used due to sparse network connections. The Surface Under the Cumulative RAnking curve (SUCRA) was used to rank the treatments.^31^ The SUCRA score takes values between 0 and 1, where higher numbers indicate higher ranked treatments. Relative effects are reported as mean and 95% credible intervals. Statistical analysis was performed using R Studio 3.6.3 with package R2WinBUGS and WINBUGS14. The model code is available in Supplementary Material 3.

### Risk of Bias

We assessed Risk of Bias (RoB) for every included study using the Cochrane risk of bias tool for randomized controlled trials.^32^ The tool evaluates 7 domains: random sequence generation, allocation concealment, blinding of participants and personnel, blinding of outcome assessment, incomplete outcome data, selective reporting and other in order to assess selection bias, performance bias, detection bias, attrition bias, and reporting bias.

Based on available information, each domain was judged by 2 independent reviewers to be of high or low risk or unclear. Disagreements were resolved in discussion.

## Results

### Selected Studies and Characteristics

**Figure 1.**
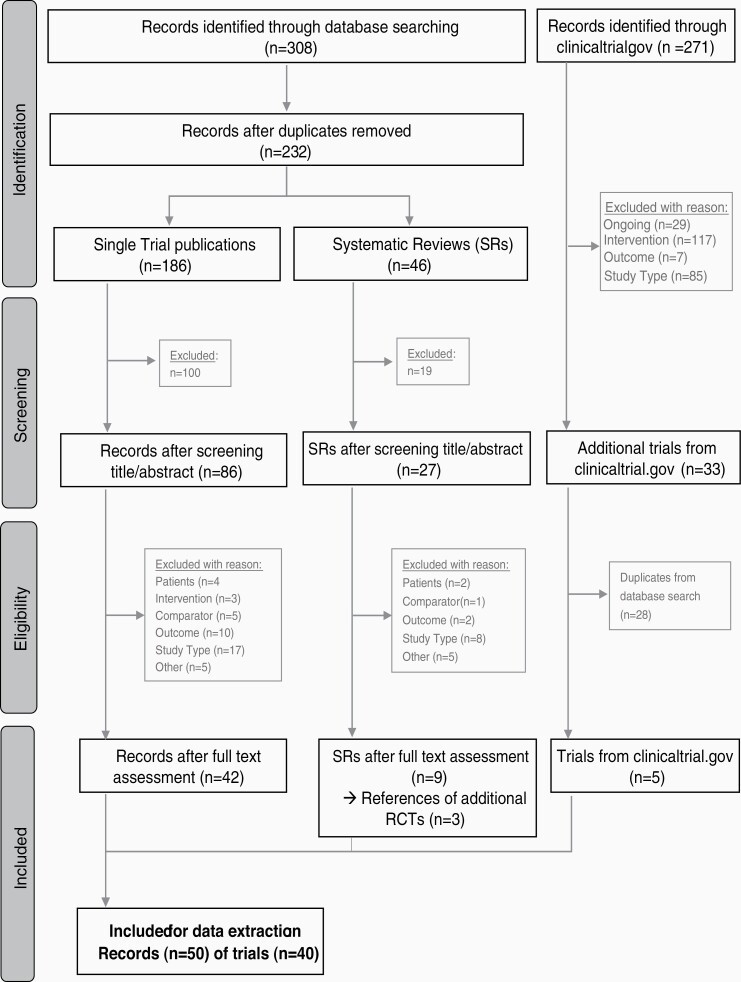
Flowchart of study selection.

A total of 308 records were identified through the database search and 271 records through clinicaltrials.gov (Figure 1). After duplicates were removed, 232 records remained from the database search. Following title and abstract screening, 86 single trial publications were available, 27 systematic reviews and 33 trials from clinicaltrials.gov. PICOS criteria were checked in full text screening, resulting in 42 records of single trial publications, 3 references of additional RCTs from systematic reviews and 5 trials from clinicaltrials.gov. A total of 50 records were included in the systematic review providing information on 40 RCTs, which fulfilled our inclusion criteria evaluating the efficacy of 56 treatment regimens for OS and 46 for PFS.

A median of 119 participants were included per study with a minimum of 21 participants and a maximum of 437. Thirty-two (80.0%) trials included 2 treatment arms and 8 (20.0%) trials included 3 treatment arms. The mean age of all study participants was 54.0 (range: 49.7–58.5) with 63.4% (range: 32.4%–78.0%) being male. The vast majority of the studies were phase II studies (30/40; 75.0%), 1 study (2.5%; not included in the NMA due to lack of connection) was a phase I study, and 9 studies were phase III studies (22.5 %). Study characteristics can be found in Supplementary Material 4.

### Efficacy

**Figure 2.**
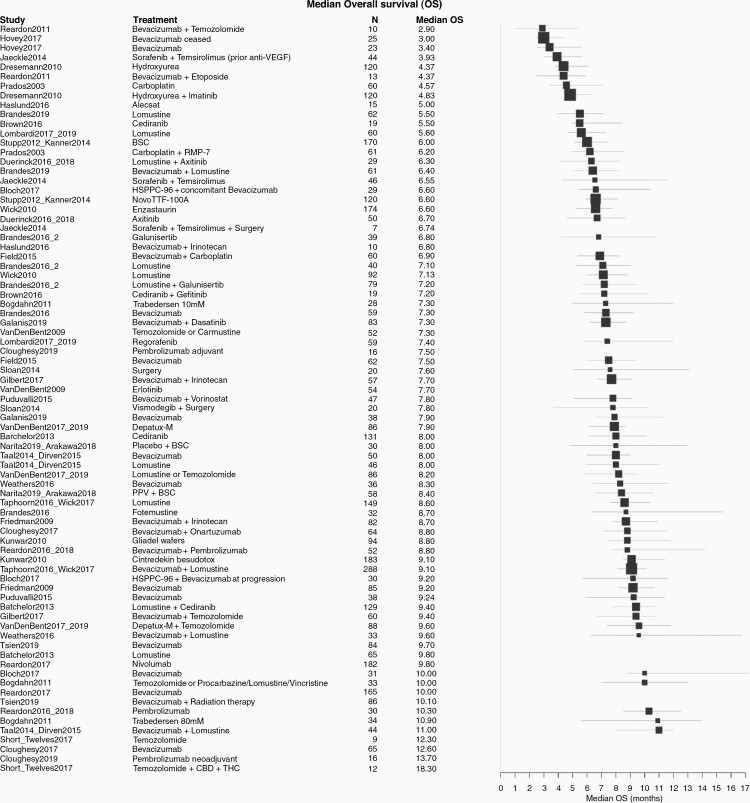
Forest plot showing trial level outcomes of overall survival. BSC, Best supportive care; CBP, cannabidiol; THC, delta-9-tetrahydrocannabinol; PPV, personal peptide vaccination.

**Figure 3.**
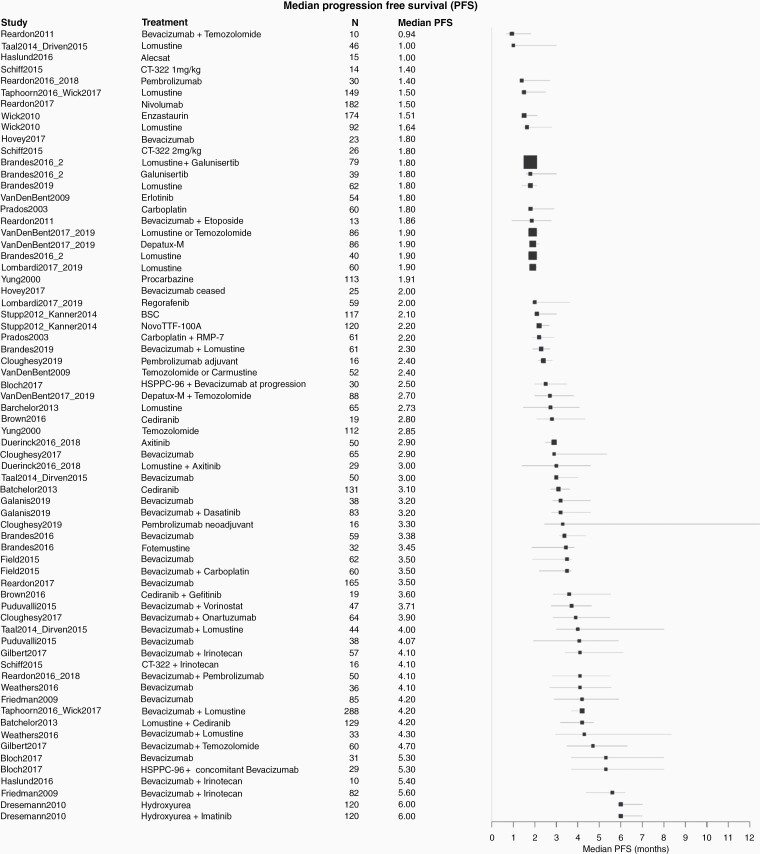
Forest plot showing trial level outcomes of progression-free survival. BSC, Best supportive care; CBP, cannabidiol; THC, delta-9-tetrahydrocannabinol; PPV, personal peptide vaccination.

Across all trials, the median OS for patients ranged from 2.9 to 18.3 months; median PFS ranged from 0.7 to 6.0 months (Figures 2 and 3). The study by Reardon et al. (2011)^33^ showed the lowest median OS with TMZ (2.9 months) which may be due to the fact that 78 % of the study participants had two or more recurrences already. In addition, the number of study participants was rather low (*n* = 10). The combination therapy of dose intense TMZ + cannabidiol (CBP) + delta-9-tetrahydrocannabinol (THC) in Short et al. (2017)^34^ and Twelves et al. (2017)^35^ had the highest median OS with 18.3 months. These results also need to be interpreted with caution, as the number of study participants was also low (*n* = 12), no confidence intervals were provided and 100% of study participants had one recurrence only, demonstrating a less sick population. The median OS for BV monotherapy ranged from 3.4 months^36^ to 12.6 months ^37^. For BV combination therapies, the median OS ranged from 6.4 months^38^ to 11.0 months^39,40^) with BV + CCNU. The lowest median PFS (0.94 months), was reported for TMZ in Reardon et al. (2011)^33^; and the highest (6.0 months) in Dresemann et al. (2010)^41^ with hydroxyurea as mono-therapy or hydroxyurea + imatinib as combination therapy. For BV monotherapy, median PFS ranged from 1.8 months^36^ to 5.3 months^42^. The median PFS for BV combination therapy ranged from 2.3 months with BV + CCNU^38^ to 5.6 months with BV + irinotecan (CPT-11) .^13^

**Figure 4.**
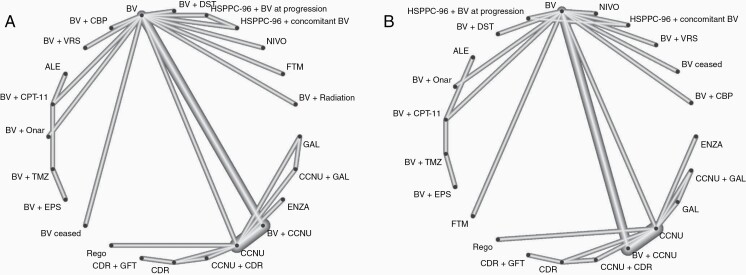
Network graph for (A) overall survival and (B) progression-free survival. Nodes indicate treatment regimen, connections between nodes indicate direct evidence comparing adjacent treatments. ALE: Alecsat, BV: Bevacizumab, CBP: Carboplatin, CCNU: Carmustine, CDR: Cediranib, CPT-11: Irinotecan, DST: Dasatinib, ENZA: Enzastaurin, EPS: Etoposide, FTM: Fotemustine, GAL: Galunisertib, GFT: Gefitinib, NIVO: Nivolumab, Onar: Onartuzumab, Rego: Regorafenib, TMZ: Temozolomide, VRS:Vorinostat.

The NMA included 24 treatment regimens for OS and 23 regimens for PFS forming a connected evidence network (Figure 4). To be able to connect more treatment regimens, we assumed equal efficacy of a treatment regardless whether a placebo was added or not (ie, treatment A and treatment A + placebo were considered equal; placebos were usually added to match the number of pills in the comparator arm). A number of regimens could, however, not be connected to the network and were excluded from the analysis: TMZ as monotherapy or in combination with BV, CBP, or THC or depatux-M (also tested as monotherapy); trabedersen, pembrolizumab as mono-therapy or in combination with BV; CT-322; hydroxyurea with or without imatinib; axitinib with or without CCNU; sorafenib with or without temsirolimus; personal peptide vaccination; cintredekin besudotox; carboplatin with or without RMP-7; vismodegib; novo TTF; semustine; erlotinib; procarbazine as well as BV in combination with TMZ or pembrolizumab.

**Figure 5.**
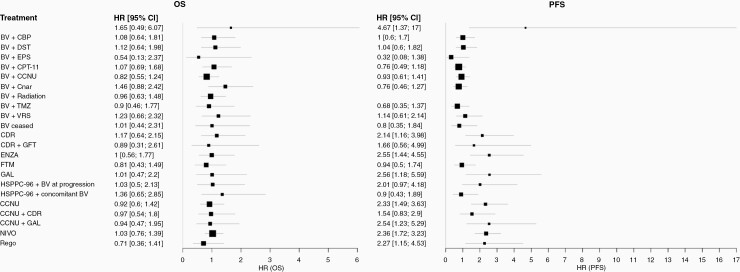
Hazard ratios (HR) of treatment comparisons versus bevacizumab. (A) For overall survival, (B) for progression-free survival. ALE: Alecsat, BV: Bevacizumab, CBP: Carboplatin, CCNU: Carmustine, CDR: Cediranib, CPT-11: Irinotecan, DST: Dasatinib, ENZA: Enzastaurin, EPS: Etoposide, FTM: Fotemustine, GAL: Galunisertib, GFT: Gefitinib, NIVO: Nivolumab, Onar: Onartuzumab, Rego: Regorafenib, TMZ: Temozolomide, VRS:Vorinostat.

**Figure 6.**
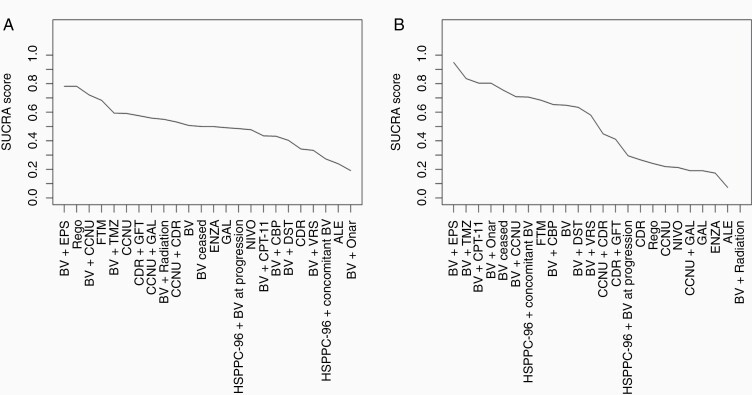
The surface under the cumulative ranking curve (SUCRA) ranking score. Higher scores indicate higher ranked treatments. (A) Overall survival and (B) progression-free survival. ALE: Alecsat, BV: Bevacizumab, CBP: Carboplatin, CCNU: Carmustine, CDR: Cediranib, CPT-11: Irinotecan, DST: Dasatinib, ENZA: Enzastaurin, EPS: Etoposide, FTM: Fotemustine, GAL: Galunisertib, GFT: Gefitinib, NIVO: Nivolumab, Onar: Onartuzumab, Rego: Regorafenib, TMZ: Temozolomide, VRS: Vorinostat.

Figure 5 displays the estimated HRs for treatment comparisons versus BV in the network; Figure 6 displays the SUCRA scores. Pairwise HRs for all treatment comparisons in the network can be found in Supplementary Material 5 for OS and 6 for PFS.

The NMA indicated no statistically significant differences with respect to OS between any of the included treatment regimens on the 95% credible level. The SUCRA score was highest for BV + etoposide (EPS) (0.78), regorafenib (0.78) and BV + CCNU (0.72) indicating that there is a higher probability that these treatment is more effective in terms of OS. On the other side, BV + onartuzumab (0.19), alecsat (0.24), and HSPPC-96 + concomitant BV (0.27) were located on the lowest ranks. BV monotherapy was located in the middle with a SUCRA score of 0.51.

More differences were observed in the analysis of PFS data. Eleven of the 12 BV-based regimen hold the top 12 ranks. The only other treatment ranked within these therapies was fotemustine (FTM) in rank 8. BV mono-therapy was ranked in 10th place. No statistically significant differences were identified between these interventions, but many of these show a statistically improved PFS when compared to regimens in the lower ranks (see Supplementary Material 6 for details). We observed no differences between the lower ranked treatment regimens.

When comparing SUCRA ranks for OS and PFS, some opposite rankings were observed. Strongest changes were observed for BV + onartuzumab ranked fourth for median PFS, and last (24) for median OS and regorafenib, which was ranked 17th (of 23) for median PFS and highest for median OS. However, as no differences between treatment regimens were identified in the OS analysis, the rank changes should not be over-interpreted.

Overall, very small benefits in survival were observed with any of the interventions, highlighting the unmet need for patients with rGBM. Our NMA indicated some improvement in PFS with most BV-based regimen compared to other regimen but we did not find any differences in OS between treatments.

### Risk of Bias

**Figure 7.**
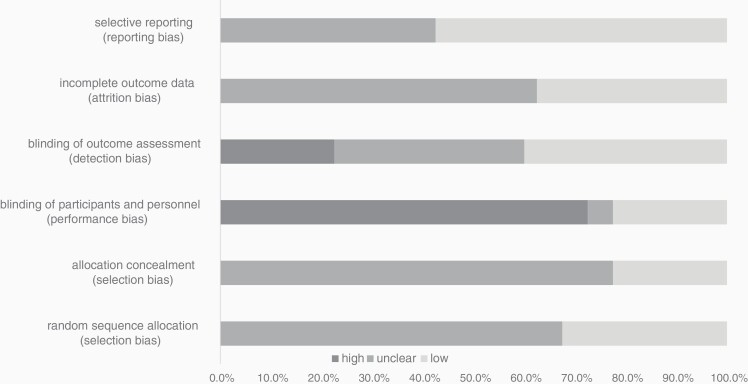
Risk of bias summary.

A risk of bias assessment was performed for each study during data extraction. See Figure 7 for a summary of the risk of bias assessment. Details per trial evaluations are displayed in Supplementary Material 7. Nonblinding of participants and personnel was declared as high risk for 72.5% of the studies. Almost all criteria showed a high percentage of unknown risk due to nonreporting.

## Discussion

We conducted an exhaustive literature review identifying a large number of RCTs evaluating the efficacy of a large number of treatment regimens for use in patients in rGBM. The most common comparator arm in the identified trials was BV, however, many trials used a different intervention and the relative efficacy between many treatment options remains unknown. To our knowledge, this study presents the largest NMA estimating relative treatment effects in rGBM conducted to date.

We found a superiority of most BV-based therapies compared to other therapy options in terms of PFS. However, this effect did not translate into an improved OS. Fotemustine showed similar efficacy in terms of PFS as BV-based therapies. No significant differences were found between treatment regimens in the analysis of OS. This finding questions the use of PFS as a surrogate outcome for OS in rGBM. While OS is the most precise and unambiguous clinical endpoint in a trial,^43^ very often, PFS is taken as the primary outcome as a surrogate for OS, as it can reduce the length of the trial and its sample size ultimately resulting in lower costs.^44^ Our findings highlight a strong need to demonstrate treatment superiority in terms of OS in rGBM trials.

Our study has some limitations.

While we were able to include a large number of trials in our NMA, not all trials contributed to a connected network. Hence, these could not be included in our analysis and no relative treatment effects could be estimated for these regimens. In the absence of connecting RCTs, additional research incorporating nonrandomized evidence or matching methods or additional assumptions on additivity of treatment components could be used to establish a connection, as has been done in the area of multiple myeloma for example.^30,45^ However, certainty in the results would suffer due to the reliance on additional assumptions for nonrandomized evidence.

There was some heterogeneity across the included studies, which the model did not account for. Included trials were conducted over a 20-year time horizon, the earliest publication dating back to 2000. The definition of progression and response changes over time, with MacDonald^46^ criteria more commonly used in earlier trials compared to the Response Assessment in Neuro-Oncology (RANO)^47,48^ more commonly used in later trials. Additionally, the number of recurrences of study participants varied across studies, with some studies only including patients experiencing first recurrence,^39,40,49–51^ 1 study^33^ including 78% of participants with 2 or more recurrences, 5 studies^13,36,52–54^ with more than 60% of participants experiencing first recurrence and less than 40% experiencing their second recurrence. Unfortunately, a high rate of included studies (14 out of 23) did not provide detailed information regarding the number of recurrences and their distribution across the included participants. Due to this low number of reporting, it was unfortunately not possible to include the number of recurrences in the statistical analysis. Further, the proportion of male participants varied from 32% to 78%, which may cause some level of heterogeneity, considering evidence that male gender is predictive of poorer outcomes. Due to the sparse network connections, we were unable to fit a random effect model or meta-regression accounting for some of this heterogeneity.

Our NMA included studies differing in size, with arms including between 10 and 288 patients. While increased uncertainty due to small trials is propagated throughout the network, some of our results still rely on very small studies only. The top ranked treatment, BV + EPS, for example, was only investigated in one small trial,^33^ including 23 patients in total, where it compared favorably (if not significantly so) with its comparator. Nevertheless, our goal was to incorporate all available evidence in the analysis, regardless of number of included patients and we feel that the uncertainty is reflected in our results.

The results of our RoB assessment indicate that there were many design flaws in the trials. Lack of blinding caused many of the trials to be of high risk of bias. Bias in study designs leads to misinterpretation of what the study outcome can demonstrate and it is often not possible to interpret the true results of these studies. In the review presented here, the large proportion of high risk of bias in at least one dimension did not allow for a subgroup analysis excluding these trials.

We chose PFS and OS as outcomes for our NMA, as they are regarded as the gold standard in oncology studies and were most widely reported across the trials. In order to make clinical decisions, other endpoints, including for instance adverse events and quality of life, also need to be taken into account. Unfortunately, these endpoints were not reported often enough or sufficiently homogenous to conduct a comparative analysis. Our NMA model relies on median PFS and OS values, as these were most widely reported in the trials. Alternative models based on reported HR assuming a normal likelihood, would have resulted in a much smaller number of trials to be included.

Despite BV not being approved as a standard of care in the treatment of rGBM, it is widely used in clinical trials, alone or combined with other drugs and therapies.

While our analysis shows no difference in any included treatment regimen with OS as an endpoint, we observed more differences with PFS and BV combination therapies ranked highest in terms of PFS. While SUCRA scores can be helpful, in terms of ranking treatments in order of probability of benefit, it should also be interpreted carefully when significant heterogeneity exists, as is the case here.^55^

The introduction of new treatments in cancer such as immunotherapy has changed the paradigm of cancer treatment.^56^ Our list of references encompasses trials based on chemotherapeutic or immunotherapy studies. The use of immunotherapy in the rGBM treatment covers many drug classes including checkpoint inhibitors (nivolumab, pembrolizumab), vaccines (HSPPC-96) or antibodies (onartuzumab, BV) and Alecsat (Autologous Lymphoid Effector Cells Specific Against Tumor Cells), a new epigenetic approach to immunotherapy. While our systematic review identified trials evaluating pembrolizumab,^57^ unfortunately, they did not connect to the evidence network and could not be included in our NMA. Our analysis validates the superiority of BV-based regimens compared to many other regimens in terms of PFS. Additional evidence is needed to derive evidence on how these interventions compare to others.

## Conclusions

Comparative treatment effects are key to guide clinical decision-making. Comparative trials between new and innovative interventions and existing treatments are needed to establish such evidence. NMA is a way of estimating relative treatment effects within a connected network of clinical trials, reducing the number of clinical trials needed to compare a large number of regimens. Our review highlighted a lack of comparative trials, which prevented us from establishing relative treatment effects between many regimens. Future trials of new interventions need to compare to existing interventions to allow for the estimation of such effects.

NMA results need to be interpreted carefully, especially where trial heterogeneity is high. Consistent reporting of important confounding variables, such as previous treatment history for example, would allow adjusting for some level of heterogeneity.

While there has been a steady publication rate of new clinical trials investigating additional treatment regimens, especially during the last 10 years, outcomes for patients with rGBM remain poor. We found no significant improvement in OS for any of the evaluated regimens compared to others. BV-based therapies demonstrated some superiority in terms of PFS.

Overall, our analysis highlights the high need to develop new and innovative treatments for this patient population delivering advances in patient relevant outcomes.

## Supplementary Material

vdab052_suppl_Supplementary_Materials

## Notes

**Authorship Statement.** Design: A.S., N.A., A.F., C.D., G.B., L.H., S.S.; Implementation: A.S., N.A., A.F., S.S.; Analysis: A.S., N.A., S.S.; Interpretation: A.S., N.A., A.F., R.A., G.B., L.H., S.S.; Writing + Revision: A.S., N.A., A.F., C.D., R.A., G.B., L.H., and S.S.

## References

[1] Gupta A , Dwivedi T. A simplified overview of world health organization classification update of central nervous system tumors 2016. J Neurosci Rural Pract. 2017; 8(4):629–641. 29204027 10.4103/jnrp.jnrp_168_17 PMC5709890

[2] Guyot P , Ades AE, Ouwens MJ, Welton NJ. Enhanced secondary analysis of survival data: reconstructing the data from published Kaplan-Meier survival curves. BMC Med Res Methodol. 2012;12:9. 22297116 10.1186/1471-2288-12-9 PMC3313891

[3] van Linde ME , Brahm CG, de Witt Hamer PC, et al. Treatment outcome of patients with recurrent glioblastoma multiforme: a retrospective multicenter analysis. J Neurooncol. 2017;135(1):183–192. 28730289 10.1007/s11060-017-2564-z PMC5658463

[4] Qazi MA , Vora P, Venugopal C, et al. Intratumoral heterogeneity: pathways to treatment resistance and relapse in human glioblastoma. Ann Oncol. 2017;28(7):1448–1456. 28407030 10.1093/annonc/mdx169

[5] Franceschi E , Bartolotti M, Brandes AA. Bevacizumab in recurrent glioblastoma: open issues. Future Oncol. 2015;11(19):2655–2665. 26357999 10.2217/fon.15.125

[6] Gorlia T , Stupp R, Brandes AA, et al. New prognostic factors and calculators for outcome prediction in patients with recurrent glioblastoma: a pooled analysis of EORTC Brain Tumour Group phase I and II clinical trials. Eur J Cancer. 2012;48(8):1176–1184. 22464345 10.1016/j.ejca.2012.02.004

[7] Abdel-Rahman O , Fouad M. Irinotecan-based regimens for recurrent glioblastoma multiforme: [corrected] a systematic review. Expert Rev Neurother. 2015;15(11):1255–1270. 26469869 10.1586/14737175.2015.1101346

[8] Kazmi F , Soon YY, Leong YH, Koh WY, Vellayappan B. Re-irradiation for recurrent glioblastoma (GBM): a systematic review and meta-analysis. J Neurooncol. 2019;142(1):79–90. 30523605 10.1007/s11060-018-03064-0

[9] Chaul-Barbosa C , Marques DF. How we treat recurrent glioblastoma today and current evidence. Curr Oncol Rep. 2019;21(10):94. 31606796 10.1007/s11912-019-0834-y

[10] Park JK , Hodges T, Arko L, et al. Scale to predict survival after surgery for recurrent glioblastoma multiforme. J Clin Oncol. 2010;28(24):3838–3843. 20644085 10.1200/JCO.2010.30.0582 PMC2940401

[11] Djamel-Eddine Y-C , De Witte O, Mélot C, Lefranc F. Recurrent glioblastomas: should we operate a second and even a third time? Interdiscip Neurosurg. 2019;18:100551.

[12] Franceschi E , Bartolotti M, Tosoni A, et al. The effect of re-operation on survival in patients with recurrent glioblastoma. Anticancer Res. 2015;35(3):1743–1748. 25750337

[13] Friedman HS , Prados MD, Wen PY, et al. Bevacizumab alone and in combination with irinotecan in recurrent glioblastoma. J Clin Oncol. 2009;27(28):4733–4740. 19720927 10.1200/JCO.2008.19.8721

[14] Cohen MH , Shen YL, Keegan P, Pazdur R. FDA drug approval summary: bevacizumab (Avastin) as treatment of recurrent glioblastoma multiforme. Oncologist. 2009;14(11):1131–1138. 19897538 10.1634/theoncologist.2009-0121

[15] Mallick S , Benson R, Hakim A, Rath GK. Management of glioblastoma after recurrence: a changing paradigm. J Egypt Natl Canc Inst. 2016;28(4):199–210. 27476474 10.1016/j.jnci.2016.07.001

[16] Egger M , Ebrahim S, Smith GD. Where now for meta-analysis? Int J Epidemiol. 2002;31(1):1–5. 11914281 10.1093/ije/31.1.1

[17] Sutton AJ , Abrams KR, Jones DR, Jones DR, Sheldon TA, Song F. Methods for meta-analysis in medical research. Vol 348: Chichester, West Sussex, United Kingdom: John Wiley & Sons, Ltd.; 2000.

[18] Lu G , Ades AE. Combination of direct and indirect evidence in mixed treatment comparisons. Stat Med. 2004;23(20):3105–3124. 15449338 10.1002/sim.1875

[19] Caldwell DM , Ades AE, Higgins JP. Simultaneous comparison of multiple treatments: combining direct and indirect evidence. BMJ. 2005;331(7521):897–900. 16223826 10.1136/bmj.331.7521.897 PMC1255806

[20] Jansen JP , Crawford B, Bergman G, Stam W. Bayesian meta-analysis of multiple treatment comparisons: an introduction to mixed treatment comparisons. Value Health. 2008;11(5):956–964. 18489499 10.1111/j.1524-4733.2008.00347.x

[21] Wong ET , Gautam S, Malchow C, Lun M, Pan E, Brem S. Bevacizumab for recurrent glioblastoma multiforme: a meta-analysis. J Natl Compr Canc Netw. 2011;9(4):403–407. 21464145 10.6004/jnccn.2011.0037

[22] de Franca SA , Tavares WM, Salinet ASM, Teixeira MJ, Paiva WS. Laser interstitial thermal therapy as an adjunct therapy in brain tumors: a meta-analysis and comparison with stereotactic radiotherapy. Surg Neurol Int. 2020;11. 10.25259/SNI_152_2020 PMC7656052 33194293

[23] Group GM-aTG. Chemotherapy in adult high-grade glioma: a systematic review and meta-analysis of individual patient data from 12 randomised trials. Lancet. 2002;359(9311):1011–1018. 11937180 10.1016/s0140-6736(02)08091-1

[24] Fu P , He YS, Huang Q, et al. Bevacizumab treatment for newly diagnosed glioblastoma: systematic review and meta-analysis of clinical trials. Mol Clin Oncol. 2016;4(5):833–838. 27123291 10.3892/mco.2016.816 PMC4840497

[25] Li M , Song X, Zhu J, Fu A, Li J, Chen T. The interventional effect of new drugs combined with the Stupp protocol on glioblastoma: a network meta-analysis. Clin Neurol Neurosurg. 2017;159:6–12. 28514722 10.1016/j.clineuro.2017.05.015

[26] Qi L , Ding L, Wang S, et al. A network meta-analysis: the overall and progression-free survival of glioma patients treated by different chemotherapeutic interventions combined with radiation therapy (RT). Oncotarget. 2016;7(42):69002–69013. 27458167 10.18632/oncotarget.10763 PMC5356607

[27] Wang Y , Chen W, Zhao B, et al. Comparative efficacy of different therapies for recurrent glioblastoma: a systematic review and Bayesian network meta-analysis. *Available at SSRN 3576919.* 2020. https://papers.ssrn.com/sol3/papers.cfm?abstract_id=3576919

[28] Moher D , Liberati A, Tetzlaff J, Altman DG; PRISMA Group. Preferred reporting items for systematic reviews and meta-analyses: the PRISMA statement. Ann Intern Med. 2009;151(4):264–9, W64. 19622511 10.7326/0003-4819-151-4-200908180-00135

[29] *WebPlotDigitizer* [computer program]. Version 4.2. San Francisco, CA; 2019. https://apps.automeris.io/wpd/

[30] Schmitz S , Maguire Á, Morris J, et al. The use of single armed observational data to closing the gap in otherwise disconnected evidence networks: a network meta-analysis in multiple myeloma. BMC Med Res Methodol. 2018;18(1):66. 29954322 10.1186/s12874-018-0509-7 PMC6022299

[31] Chaimani A , Higgins JP, Mavridis D, Spyridonos P, Salanti G. Graphical tools for network meta-analysis in STATA. PLoS One. 2013;8(10):e76654. 24098547 10.1371/journal.pone.0076654 PMC3789683

[32] Higgins JP , Altman DG, Gøtzsche PC, et al. The cochrane collaboration’s tool for assessing risk of bias in randomised trials. BMJ. 2011;343:d5928. 22008217 10.1136/bmj.d5928 PMC3196245

[33] Reardon DA , Desjardins A, Peters K, et al. Phase II study of metronomic chemotherapy with bevacizumab for recurrent glioblastoma after progression on bevacizumab therapy. J Neurooncol. 2011;103(2):371–379. 20853132 10.1007/s11060-010-0403-6 PMC3102515

[34] Short SC , Little C. ACTR-56: a 2-part safety and exploratory efficacy randomised double-blind, placebo-controlled study of a 1:1 ratio of cannabidiol and delta9-tetrahydrocannabinol (CBD:THC) plus doseintense temozolomide in patients with recurrent glioblastoma multiforme (GBM). Neuro-Oncology; Abstracts from the 22nd Annual Scientific Meeting and Education Day of the Society for Neuro-Oncology November 16 – 19, 2017, San Francisco, California. 2017; 19(suppl_6):vi1–vi314.

[35] Twelves C , Short S, Wright S. A two-part safety and exploratory efficacy randomized double-blind, placebo-controlled study of a 1:1 ratio of the cannabinoids cannabidiol and delta-9-tetrahydrocannabinol (CBD: THC) plus dose-intense temozolomide in patients with recurrent glioblastoma multiforme (GBM). J Clin Oncol. 2017;35(15):2046–2046.

[36] Hovey EJ , Field KM, Rosenthal MA, et al. Continuing or ceasing bevacizumab beyond progression in recurrent glioblastoma: an exploratory randomized phase II trial. Neurooncol Pract. 2017;4(3):171–181. 31386014 10.1093/nop/npw025 PMC6655481

[37] Cloughesy T , Finocchiaro G, Belda-Iniesta C, et al. Randomized, double-blind, placebo-controlled, multicenter phase II study of onartuzumab plus bevacizumab versus placebo plus bevacizumab in patients with recurrent glioblastoma: efficacy, safety, and hepatocyte growth factor and O(6)-Methylguanine-DNA methyltransferase biomarker analyses. J Clin Oncol. 2017;35(3):343–351. 27918718 10.1200/JCO.2015.64.7685

[38] Brandes AA , Gil-Gil M, Saran F, et al. A randomized Phase II trial (TAMIGA) evaluating the efficacy and safety of continuous bevacizumab through multiple lines of treatment for recurrent Glioblastoma. Oncologist. 2019;24(4):521–528. 30266892 10.1634/theoncologist.2018-0290 PMC6459244

[39] Taal W , Oosterkamp HM, Walenkamp AM, et al. Single-agent bevacizumab or lomustine versus a combination of bevacizumab plus lomustine in patients with recurrent glioblastoma (BELOB trial): a randomised controlled phase 2 trial. Lancet Oncol. 2014;15(9):943–953. 25035291 10.1016/S1470-2045(14)70314-6

[40] Dirven L , van den Bent MJ, Bottomley A, et al. The impact of bevacizumab on health-related quality of life in patients treated for recurrent glioblastoma: results of the randomised controlled phase 2 BELOB trial. Eur J Cancer. 2015;51(10):1321–1330. 25899986 10.1016/j.ejca.2015.03.025

[41] Dresemann G , Weller M, Rosenthal MA, et al. Imatinib in combination with hydroxyurea versus hydroxyurea alone as oral therapy in patients with progressive pretreated glioblastoma resistant to standard dose temozolomide. J Neurooncol. 2010;96(3):393–402. 19688297 10.1007/s11060-009-9976-3

[42] Bloch O , Shi Q, Anderson SK, et al. ATIM-14. ALLIANCE A071101: a phase II randomized trial comparing the efficacy of heat shock protein peptide Complex-96 (HDPPC-96) vaccine given with bevacizumab versus bevacizumab alone in the treatment of surgically resectable recurrent glioblastoma. Neuro-Oncology; Abstracts from the 22nd Annual Scientific Meeting and Education Day of the Society for Neuro-Oncology November 16 – 19, 2017, San Francisco, California. 2017;19(suppl_6):vi1–vi314.

[43] Clinical Trial Endpoints for the Approval of Cancer Drugs and Biologics Guidance for Industry. U.S. Department of Health and Human ServicesFood and Drug Administration Oncology Center of ExcellenceCenter for Drug Evaluation and Research (CDER)Center for Biologics Evaluation and Research (CBER). Clinical/Medical; December 2018.

[44] Gutman SI , Piper M, Grant MD, Basch E, Oliansky DM, Aronson N. Progression-Free Survival: What Does it Mean for Psychological Well-Being or Quality of Life ? Rockville, MD: Agency for Healthcare Research and Quality (US); 2013. 23678517

[45] Rücker G , Schmitz S, Schwarzer G. Component network meta‐analysis compared to a matching method in a disconnected network: a case study. Biometrical Journal. 2020;63(2):447–461. 32596834 10.1002/bimj.201900339

[46] Macdonald DR , Cascino TL, Schold SC Jr, Cairncross JG. Response criteria for phase II studies of supratentorial malignant glioma. J Clin Oncol. 1990;8(7):1277–1280. 2358840 10.1200/JCO.1990.8.7.1277

[47] Wen PY , Chang SM, Van den Bent MJ, Vogelbaum MA, Macdonald DR, Lee EQ. Response assessment in neuro-oncology clinical trials. J Clin Oncol. 2017;35(21):2439–2449. 28640707 10.1200/JCO.2017.72.7511 PMC5516482

[48] Wen PY , Macdonald DR, Reardon DA, et al. Updated response assessment criteria for high-grade gliomas: response assessment in neuro-oncology working group. J Clin Oncol. 2010;28(11):1963–1972. 20231676 10.1200/JCO.2009.26.3541

[49] Reardon DA , Omuro A, Brandes AA, et al. OS10.3: randomized phase 3 study evaluating the efficacy and safety of Nivolumab vs Bevacizumab in patients with recurrent glioblastoma: checkmate 143. Neuro-Oncology; 5th Quadrennial Meeting of the World Federation of Neuro-Oncology Societies (WFNOS). 2017; 19(suppl_3):iii21–iii21.

[50] Lombardi G , De Salvo GL, Brandes AA, et al. LBA16: REGOMA: a randomized, multicenter, controlled open-label phase II clinical trial evaluating regorafenib activity in relapsed glioblastoma patients. Annals of Oncology; Abstract Book of the 42nd ESMO Congress (ESMO 2017) 8–12 September 2017, Madrid, Spain. 2017; 28:v610.

[51] Lombardi G , De Salvo GL, Brandes AA, et al. Regorafenib compared with lomustine in patients with relapsed glioblastoma (REGOMA): a multicentre, open-label, randomised, controlled, phase 2 trial. Lancet Oncol. 2019;20(1):110–119. 30522967 10.1016/S1470-2045(18)30675-2

[52] Wick W , Puduvalli VK, Chamberlain MC, et al. Phase III study of enzastaurin compared with lomustine in the treatment of recurrent intracranial glioblastoma. J Clin Oncol. 2010;28(7):1168–1174. 20124186 10.1200/JCO.2009.23.2595 PMC2834468

[53] Field KM , Simes J, Nowak AK, et al. Randomized phase 2 study of carboplatin and bevacizumab in recurrent glioblastoma. Neuro Oncol. 2015;17(11):1504–1513. 26130744 10.1093/neuonc/nov104 PMC4648304

[54] Weathers SP , Han X, Liu DD, et al. A randomized phase II trial of standard dose bevacizumab versus low dose bevacizumab plus lomustine (CCNU) in adults with recurrent glioblastoma. J Neurooncol. 2016;129(3):487–494. 27406589 10.1007/s11060-016-2195-9 PMC5021605

[55] Mbuagbaw L , Rochwerg B, Jaeschke R, et al. Approaches to interpreting and choosing the best treatments in network meta-analyses. J Syst Rev. 2017;6(1):1–5. 10.1186/s13643-017-0473-z PMC5389085 28403893

[56] Dobosz P , Dzieciątkowski T. The intriguing history of cancer immunotherapy. Front Immunol. 2019;10:2965. 31921205 10.3389/fimmu.2019.02965 PMC6928196

[57] Cloughesy TF , Mochizuki AY, Orpilla JR, et al. Neoadjuvant anti-PD-1 immunotherapy promotes a survival benefit with intratumoral and systemic immune responses in recurrent glioblastoma. Nat Med. 2019;25(3):477–486. 30742122 10.1038/s41591-018-0337-7 PMC6408961

