# Supplementary material for: Systematic review and network meta-analysis of the efficacy of existing treatments for patients with recurrent glioblastoma
Source: Neurooncol Adv. 2021 Apr 9;3(1):vdab052. doi: 10.1093/noajnl/vdab052 (PMC8174573; doi:10.1093/noajnl/vdab052)
Supplement: vdab052_suppl_Supplementary_Materials [file vdab052_suppl_supplementary_materials.zip › Supplementary_Material_S7.docx]

| Supp. 7: Risk of Bias |  |  |  |  |  |  |  |  |  |  |
| --- | --- | --- | --- | --- | --- | --- | --- | --- | --- | --- |
|  | random sequence  allocation (selection bias) | allocation concealment (selection bias) | blinding of participants  and personnel (performance bias) | blinding of outcome assessment (detection bias) | incomplete outcome  data (attrition bias) | selective reporting (reporting bias) |  |  |  |  |
| Batchelor 2013 | ? | ? | high | low | ? | ? |  |  |  |  |
| Bloch 2017 | ? | ? | ? | ? | ? | ? |  |  |  |  |
| Bogdahn 2011 | ? | low | high | low | low | ? |  |  |  |  |
| Brandes 2016 | ? | ? | high | ? | low | low |  |  |  |  |
| Brandes, Carpentier 2016 | low | low | low | low | low | ? |  |  |  |  |
| Brandes 2019 | ? | ? | low | ? | ? | ? |  |  |  |  |
| Brown 2016 | low | low | low | ? | low | low |  | low | low risk of bias | |
| Cloughesy 2017 | low | ? | low | ? | ? | low |  | ? | unknown risk of bias | |
| Cloughesy 2019 | ? | ? | high | high | ? | low |  | high | high risk of bias | |
| Dresemann 2010 | ? | ? | high | low | low | low |  |  |  |  |
| Duerinck 2018 | ? | ? | high | high | low | low |  |  |  |  |
| Field 2015 | ? | ? | high | low | low | low |  |  |  |  |
| Friedman 2009 | ? | ? | high | low | low | low |  |  |  |  |
| Galanis 2015 | ? | ? | low | low | ? | ? |  |  |  |  |
| Galanis 2017, 2019 | ? | ? | low | low | low | low |  |  |  |  |
| Gilbert 2017 | low | ? | low | low | ? | low |  |  |  |  |
| Haslund 2016 | ? | ? | low | ? | ? | ? |  |  |  |  |
| Hovey 2017 | ? | ? | high | high | low | low |  |  |  |  |
| Jaeckle 2014 | ? | ? | high | ? | ? | low |  |  |  |  |
| Kunwar 2010 | ? | ? | high | low | ? | ? |  |  |  |  |
| Lombardi 2017, 2019 | low | low | high | high | low | low |  |  |  |  |
| Narita 2019 | low | low | low | ? | low | low |  |  |  |  |
| Prados 2003 | low | low | low | low | ? | ? |  |  |  |  |
| Puduvalli 2015 | low | ? | high | ? | ? | low |  |  |  |  |
| Reardon 2011 | ? | ? | high | high | low | ? |  |  |  |  |
| Reardon 2016, 2018 | ? | ? | high | ? | ? | ? |  |  |  |  |
| Reardon 2017 | ? | ? | high | ? | ? | low |  |  |  |  |
| Schiff 2015 | ? | ? | high | ? | ? | ? |  |  |  |  |
| Short, Twelves 2017 | ? | ? | low | low | ? | low |  |  |  |  |
| Sloan 2014 | ? | ? | low | ? | ? | low |  |  |  |  |
| Stupp 2012, Kanner 2014 | low | ? | high | low | ? | low |  |  |  |  |
| Sun 2013 | ? | ? | high | ? | ? | low |  |  |  |  |
| Taal 2014, Dirven 2015 | low | low | high | high | ? | ? |  |  |  |  |
| Taphoorn 2016, Wick 2017 | low | low | high | high | ? | ? |  |  |  |  |
| Tsien 2019 | low | ? | high | high | low | low |  |  |  |  |
| VanDenBent 2009 | ? | low | high | ? | ? | low |  |  |  |  |
| VanDenBent 2017, 2019 | low | ? | high | low | low | low |  |  |  |  |
| Weathers 2016 | ? | ? | ? | low | ? | ? |  |  |  |  |
| Wick 2010 | ? | ? | high | low | ? | ? |  |  |  |  |
| Yung 2000 | ? | ? | high | low | ? | ? |  |  |  |  |
