# Supplementary material for: Systematic review and network meta-analysis of the efficacy of existing treatments for patients with recurrent glioblastoma
Source: Neurooncol Adv. 2021 Apr 9;3(1):vdab052. doi: 10.1093/noajnl/vdab052 (PMC8174573; doi:10.1093/noajnl/vdab052)
Supplement: vdab052_suppl_Supplementary_Materials [file vdab052_suppl_supplementary_materials.zip › Supplementary_Material_S1.docx]

## Supp. 1:

## Search strategy

We searched EMBASE, MEDLINE and CENTRAL to identify randomised controlled trials (RCTs) excluding phase I trials, as well as systematic reviews including RCTs on adult patients with recurrent glioblastoma (rGBM) receiving any treatment compared to either placebo or an active comparator, which report efficacy outcomes including overall survival or progression free survival.

We first comprised all terms or synonyms used to define rGBM as keywords in our search strategy. Keywords included were recurrent glioblastoma, rglioblastoma, rGBM, Astrocytoma grade IV, Astrocytomas grade IV, grade IV Astrocytoma, grade IV Astrocytomas, Recurrent Grade IV Astrocytoma and Recurrent High-grade IV Gliomas.

Our search was not limited in terms of intervention, comparator and outcome definitions.

We limited the search to articles reporting results of an RCT or articles presenting a systematic review of RCTs in rGBM:

Relevant RCTs were identified by limiting the search of rGBM articles to RCTs using a filter and a keyword approach, and simultaneously excluding phase I trials, also using a filter and a keyword approach.

Systematic reviews were identified by limiting the search of rGBM articles to systematic reviews using a filter as well as a keyword approach.

The complete search strategy as applied for EMBASE can be found below. The same strategy was followed in all databases.

| ((("recurrent glioblastoma"[Text Word]) OR (rglioblastoma[Text Word]) OR (rglioblastoma[Text Word]) OR (rGBM[Text Word]) OR ("Astrocytoma grade IV"[Text Word] OR "Astrocytomas grade IV"[Text Word] OR "grade IV Astrocytoma"[Text Word] OR "grade IV Astrocytomas"[Text Word]) OR (Recurrent Grade IV Astrocytoma[Text Word]) OR (Recurrent High-grade IV Gliomas[Text Word]) AND (systematic[sb])) OR ((("recurrent glioblastoma"[Text Word]) OR (rglioblastoma[Text Word]) OR (rglioblastoma[Text Word]) OR (rGBM[Text Word]) OR ("Astrocytoma grade IV"[Text Word] OR "Astrocytomas grade IV"[Text Word] OR "grade IV Astrocytoma"[Text Word] OR "grade IV Astrocytomas"[Text Word]) OR (Recurrent Grade IV Astrocytoma[Text Word]) OR (Recurrent High-grade IV Gliomas[Text Word])) AND ("systematic review"[Text Word] OR "systematic reviews"[Text Word]))) OR (((("recurrent glioblastoma"[Text Word]) OR (rglioblastoma[Text Word]) OR (rglioblastoma[Text Word]) OR (rGBM[Text Word]) OR ("Astrocytoma grade IV"[Text Word] OR "Astrocytomas grade IV"[Text Word] OR "grade IV Astrocytoma"[Text Word] OR "grade IV Astrocytomas"[Text Word]) OR (Recurrent Grade IV Astrocytoma[Text Word]) OR (Recurrent High-grade IV Gliomas[Text Word])) NOT (("recurrent glioblastoma"[Text Word]) OR (rglioblastoma[Text Word]) OR (rglioblastoma[Text Word]) OR (rGBM[Text Word]) OR ("Astrocytoma grade IV"[Text Word] OR "Astrocytomas grade IV"[Text Word] OR "grade IV Astrocytoma"[Text Word] OR "grade IV Astrocytomas"[Text Word]) OR (Recurrent Grade IV Astrocytoma[Text Word]) OR (Recurrent High-grade IV Gliomas[Text Word]) AND (Clinical Trial, Phase I[ptyp])) AND (Randomized Controlled Trial[ptyp])) OR (((("recurrent glioblastoma"[Text Word]) OR (rglioblastoma[Text Word]) OR (rglioblastoma[Text Word]) OR (rGBM[Text Word]) OR ("Astrocytoma grade IV"[Text Word] OR "Astrocytomas grade IV"[Text Word] OR "grade IV Astrocytoma"[Text Word] OR "grade IV Astrocytomas"[Text Word]) OR (Recurrent Grade IV Astrocytoma[Text Word]) OR (Recurrent High-grade IV Gliomas[Text Word])) NOT (("recurrent glioblastoma"[Text Word]) OR (rglioblastoma[Text Word]) OR (rglioblastoma[Text Word]) OR (rGBM[Text Word]) OR ("Astrocytoma grade IV"[Text Word] OR "Astrocytomas grade IV"[Text Word] OR "grade IV Astrocytoma"[Text Word] OR "grade IV Astrocytomas"[Text Word]) OR (Recurrent Grade IV Astrocytoma[Text Word]) OR (Recurrent High-grade IV Gliomas[Text Word]) AND (Clinical Trial, Phase I[ptyp]))) AND (RCT[Text Word] OR "Randomised controlled trial"[Text Word] OR "Randomised controlled trials"[Text Word] OR "Randomized controlled trial"[Text Word] OR "Randomized controlled trials"[Text Word]))) |
| --- |

Figure 1: Complete Search Strategy for EMBASE.
