# Supplementary material for: Systematic review and network meta-analysis of the efficacy of existing treatments for patients with recurrent glioblastoma
Source: Neurooncol Adv. 2021 Apr 9;3(1):vdab052. doi: 10.1093/noajnl/vdab052 (PMC8174573; doi:10.1093/noajnl/vdab052)
Supplement: vdab052_suppl_Supplementary_Materials [file vdab052_suppl_supplementary_materials.zip › Supplementary_Material_S2.pdf]

Data collection form for recurrent Glioblastoma trial (PFP2)

Intervention review – RCTs

Suggested citation: Effective Practice and Organisation of Care (EPOC). Data collection form. EPOC Resources for review authors. Oslo: Norwegian Knowledge Centre for the Health Services; 2013. Available at: *http://epoc.cochrane.org/epoc-specific-resources-review-authors*

Notes on using a data extraction form:

- Be consistent in the order and style you use to describe the information for each included study.
- For transforming of days/ weeks/ months use:
  - Average of 30 days per month
  - Average of 4.35 weeks per month
- Record any missing information as unclear or not described, to make it clear that the information was not found in the study report(s), not that you forgot to extract it.
  - **"999" (not available) in all fields**
  - **All fields need to be filled out**
- Name final study document: "Initial_StudyID" e.g. "AS_Smith2001.pdf" and save all data extraction documents in one folder

**Review title or ID** *(ID from title and abstract screening excel file* ***ID+NR****)*
ID

**Study ID** *(surname of first author and year first full report of study was published e.g. Smith2001)*

**Trial ID** *(official trial ID)* **and trial acronym**
ID<br>Source 999
Acronym<br>Source 999

**Report IDs of other reports of this study** *(e.g. duplicate publications, follow-up studies)*
Notes:

1. General Information

1. **Date form completed** *(mm/dd/yyyy)*	
2. **Name/ID of person extracting data**	Other
3. **Report title** *(title of paper/ abstract/ report that data are extracted from)*	
4. **Report ID** *(if there are multiple reports of this study)*	
5. **Reference details** *(Name of journal etc.)*	
6. **Year of publishing**	
7. **Publication type** *(e.g. full report, abstract, letter)*	999
8. **Study funding source** *(including role of funders)*	
**Possible conflicts of interest** *(for study authors)*	
9. **Notes:**	

2. Eligibility

**Study Characteristics**	**Review Inclusion Criteria** *(Insert inclusion criteria for each characteristic as defined in the Protocol)*	**Yes/ No / Unclear**
10. **Type of study**	Randomised trial	Unclear
	Controlled study	Unclear
11. **Patients**	Adults (≥ 18 years) suffering from recurrent glioblastoma	Unclear
12. **Types of outcome measures reported**	OS Secondary O; PFS Secondary O; QoL Secondary O; Tumour response Secondary O; Safety Secondary O; Other Secondary O	Unclear
13. **Decision of inclusion**	No	
14. **Reason for exclusion**	Patients ◯ Intervention ◯ Comparator ◯ Outcome ◉ Study Type ◯ Other ◯ no exclusion ◯	
15. **Notes:**		

DO NOT PROCEED IF STUDY EXCLUDED FROM REVIEW

3. Population and setting

	**Description** *Include comparative information for each group (i.e. intervention and controls) if available*
16. **Population description** *(from which study participants are drawn)*	
17. **Setting** *(including location and social context)*	
18. **Inclusion criteria of study**	
19. **Exclusion criteria of study**	
20. **Method/s of recruitment of participants**	
21. **Notes:**	

4. Methods

	**Descriptions as stated in report/paper**			
22. **Aim of study**	Efficacy ☐	Safety ☐	Dose finding ☐	Other ☐
23. **Design** *(e.g. RCT, parallel, crossover, other)*	999			
24. **Start date of study** *(mm/YYYY)*				
25. **End date of study** *(mm/YYYY)*				
26. **Duration of participation** *(if start and end date not given; from recruitment to last follow-up, weeks)*				
27. **Analysis population** *(ITT or PP)*	999			
28. **Randomization ratio**				
29. **Notes:**				

5. Risk of Bias assessment

See Chapter 8 of the Cochrane Handbook. Additional domains may be required for non-randomised studies.

Domain	Risk of bias<br>*Low/ High/Unclear*	Support for judgement
30. **Random sequence generation**<br>*(selection bias)*	Unclear	
31. **Allocation concealment**<br>*(selection bias)*	Unclear	
32. **Blinding of participants and personnel**<br>*(performance bias)*	Unclear	
33. **Blinding of outcome assessment**<br>*(detection bias)*	Unclear	
34. **Incomplete outcome data**<br>*(attrition bias)*	Unclear	
35. **Selective outcome reporting?**<br>*(reporting bias)*	Unclear	
36. **Other bias**	Unclear	
37. **Notes:**		

6. Trial groups

**Number of groups**	

Comparator

	Description as stated in report/paper
38. **Group name**	
39. **Description**<br>*(include sufficient detail for replication, e.g. content, dose, components; if it is a natural experiment, describe the pre-intervention)*	
40. **Dose modification allowed?**	Unknown
41. **Duration of treatment period**<br>*(weeks of actual treatment; length of cycle (weeks), number of cycles)*	Mean SD<br>Median IQR: lower upper<br>Length of cycle Number of cycles<br>Until:

	**Description as stated in report/paper**
42. **Actual daily dose**	Mean SD<br>Median<br>Unit
43. **Co-interventions** *(Steroids, treatments of side effects, etc.)*	
44. **Notes:**	

Intervention Group 1

	**Description as stated in report/paper**
45. **Group name**	
46. **Description** *(include sufficient detail for replication, e.g. content, dose, components; if it is a natural experiment, describe the pre-intervention)*	
47. **Dose modification allowed?**	Unknown
48. **Duration of treatment period** *(weeks of actual treatment; length of cycle (weeks), number of cycles)*	Mean SD<br>Median IQR: lower upper<br>Length of cycle Number of cycles<br>Until:
49. **Actual daily dose**	Mean SD<br>Median<br>Unit
50. **Co-interventions** *(Steroids, treatments of side effects, etc.)*	
51. **Notes:**	

Intervention Group 2

	**Description as stated in report/paper**
52. **Group name**	
53. **Description** *(include sufficient detail for replication, e.g. content, dose, components; if it is a natural experiment, describe the pre-intervention)*	
54. **Dose modification allowed?**	Unknown
55. **Duration of treatment period** *(weeks of actual treatment; length of cycle (weeks), number of cycles)*	Mean SD<br>Median IQR: lower upper<br>Length of cycle Number of cycles<br>Until:
56. **Actual daily dose**	Mean SD<br>Median<br>Unit
57. **Co-interventions** *(Steroids, treatments of side effects, etc.)*	
58. **Notes:**	

7. Participants

	Description as stated in report/paper			
	**Total**	**Comparator**	**Intervention 1**	**Intervention 2**
**Group names**				
59. **Total N randomised**				
60. **Baseline imbalances?**	999			
61. **N withdrawals and exclusions** *(after randomization until end of study)*				
62. **Age**	Mean<br>SD<br>**95% CI:**<br>Lower<br>Upper<br>Median<br>**IQR:**<br>lower<br>upper<br>**Range:**<br>lower<br>Upper	Mean<br>SD<br>**95% CI:**<br>Lower<br>Upper<br>Median<br>**IQR:**<br>lower<br>upper<br>**Range**:<br>lower<br>Upper	Mean<br>SD<br>**95% CI:**<br>Lower<br>Upper<br>Median<br>**IQR:**<br>lower<br>upper<br>**Range**:<br>lower<br>Upper	Mean<br>SD<br>**95% CI:**<br>Lower<br>Upper<br>Median<br>**IQR:**<br>lower<br>upper<br>**Range**:<br>lower<br>Upper
63. **Sex** *(% male)*				
64. **Time since diagnosis** *(months)*	Mean<br>Median	Mean<br>Median	Mean<br>Median	Mean<br>Median
65. **Diagnosis of recurrence** *(%)*				
**1rst recurrence**				
**2nd recurrence**				
**3rd recurrence**				
66. **Performance status** *(ECOG, KPS, WHO)*	**ECOG** (%)<br>1<br>2<br>3<br>4<br>5	**ECOG** (%)<br>1<br>2<br>3<br>4<br>5	**ECOG** (%)<br>1<br>2<br>3<br>4<br>5	**ECOG** (%)<br>1<br>2<br>3<br>4<br>5
	**KPS**<br>Mean<br>Median	**KPS**<br>Mean<br>Median	**KPS**<br>Mean<br>Median	**KPS**<br>Mean<br>Median

	Description as stated in report/paper			
	**Total**	**Comparator**	**Intervention 1**	**Intervention 2**
**Group names**				
	**WHO** (%)<br>1<br>2<br>3<br>4<br>5	**WHO** (%)<br>1<br>2<br>3<br>4<br>5	**WHO** (%)<br>1<br>2<br>3<br>4<br>5	**WHO** (%)<br>1<br>2<br>3<br>4<br>5
67. **Re-surgery** *(%)*				
68. **Tumour size**	Mean<br>Median<br>Unit 999<br>Time 999	Mean<br>Median<br>Unit 999<br>Time 999	Mean<br>Median<br>Unit 999<br>Time 999	Mean<br>Median<br>Unit 999<br>Time 999
69. **MGMT promoter** *(proportion of methylated promoter)*				
70. **Steroid use** *(%)*				
71. **Median follow-up time** *(months)*<br>*if given in weeks: months = weeks/4.35*<br>*if given in days months = days/30*	**IQR**:<br>lower<br>upper<br>Method	**IQR**:<br>lower<br>upper<br>Method	**IQR**:<br>lower<br>upper<br>Method	**IQR**:<br>lower<br>upper<br>Method
72. **Notes:**				

8. Outcomes

Overall survival

	Description as stated in report/paper
73. **Outcome name** *(OS for overall survival)*	
74. **Time points measured** *(specify whether from start of intervention, after randomization, or date of diagnosis)*	999
75. **Outcome definition** *(median survival, survival rates, hazard ratio, adjusted Cox regression etc.))*	
76. **Imputation of missing data** *(e.g. assumptions made for ITT analysis)*	
77. **Notes:**	

Progression free survival

	Description as stated in report/paper
78. **Outcome name** *(PFS for progression free survival)*	
79. **Time points measured** *(specify whether from start of intervention, after randomization, or date of diagnosis)*	999
80. **Outcome definition** *(Median PFS, PFS rates, hazard ratio, adjusted Cox regression etc.))*	
81. **Imputation of missing data** *(e.g. assumptions made for ITT analysis)*	
82. **Notes:**	

Response Rate

	Description as stated in report/paper
83. **Outcome name** *(CR= complete response, OVR = overall response, PR = partial response, OR = objective response, SDI = stable disease)*	If multiple are mentioned, choose the one that is most general; e.g. overall response instead of partial response
84. **Outcome definition**	
85. **Imputation of missing data** *(e.g. assumptions made for ITT analysis)*	
86. **Notes:**	

Quality of Life

	Description as stated in report/paper
87. **Outcome name** *QoL for Quality of Life)*	
88. **Time points measured** *(specify whether from start of intervention, after randomization, or date of diagnosis)*	999
89. **Outcome definition** *(mean/median QoL, questionnaire to assess QoL, hazard ratio, adjusted Cox regression etc.))*	
90. **Imputation of missing data** *(e.g. assumptions made for ITT analysis)*	
91. **Notes:**	

9. Results

Overall Survival

	Description as stated in report/paper								
92. **Results**	**Comparison**			**Intervention 1**			**Intervention 2**		
Note whether: ○ *post-intervention OR* ○ *change from baseline* ○ *Unknown* *And whether* ○ *Adjusted OR* ○ *Unadjusted* ○ *Unknown*	Median (months)	Range	N	Median (months)	Range	N	Median (months)	Range	N
		Min<br>Max<br>**95% CI**:<br>Lower<br>Upper<br>**IQR**:<br>Lower<br>Upper			Min<br>Max<br>**95% CI:**<br>Lower<br>Upper<br>**IQR**:<br>Lower<br>Upper			Min<br>Max<br>**95% CI:**<br>Lower<br>Upper<br>**IQR**:<br>Lower<br>Upper	
**Rates**	**Comparison**			**Intervention 1**			**Intervention 2**		
	%	95% CI	N	%	95% CI	N	%	95% CI	N
6- months survival		Lower<br>Upper			Lower<br>Upper			Lower<br>Upper	

	Description as stated in report/paper								
1-year survival (%)		Lower<br>Upper			Lower<br>Upper			Lower<br>Upper	
2-year survival (%)		Lower<br>Upper			Lower<br>Upper			Lower<br>Upper	
3-year survival (%)		Lower<br>Upper			Lower<br>Upper			Lower<br>Upper	
Survival (%)		Lower<br>Upper			Lower<br>Upper			Lower<br>Upper	
93. **HR (for death)**<br>Int1 vs Comp<br><br>Int2 vs Comp	95% CI: Lower Upper p-value Test<br><br>95% CI: Lower Upper p-value Test								
94. **N missing participants and reasons** (e.g. lost to follow-up total)									
95. **N participants moved from other group and reasons (crossover)**									
96. **Any other/additional results reported?**	999<br>Kaplan- Meier Curve ☐<br>Table ☐								
97. **Statistical methods used and appropriateness of these methods** *(e.g. adjustment for correlation)*									
98. **Notes:**									

Progression free survival

	Description as stated in report/paper								
99. **Results**<br>*Note whether:*<br>○ *post-intervention OR*<br>○ *change from baseline*<br>○ *Unknown*<br>*And whether*<br>○ *Adjusted OR*<br>○ *Unadjusted*<br>○ *Unknown*	**Comparison**			**Intervention 1**			**Intervention 2**		
	Median (months)	Range	N	Median (months)	Range	N	Median (months)	Range	N
		Min<br>Max<br>**95% CI**:<br>Lower<br>Uppe<br>**IQR**:<br>Lowe<br>Uppe			Min<br>Max<br>**95% CI**:<br>Lower<br>Upper<br>**IQR**:<br>Lower<br>Upper			Min<br>Max<br>**95% CI**:<br>Lower<br>Uppe<br>**IQR**:<br>Lower<br>Upper	
100. **Rates**	**Comparison**			**Intervention 1**			**Intervention 2**		
	%	95% CI	N	%	95% CI	N	%	95% CI	N
6- months PFS (%)		Lower<br>Upper			Lower<br>Upper			Lower<br>Upper	
1-year PFS (%)		Lower<br>Upper			Lower<br>Upper			Lower<br>Upper	
2-year PFS (%)		Lower<br>Upper			Lower<br>Upper			Lower<br>Upper	
3-year PFS (%)		Lower<br>Upper			Lower<br>Upper			Lower<br>Upper	
PFS (%)		Lower<br>Upper			Lower<br>Upper			Lower<br>Upper	
101. **HR**<br>Int1 vs Comp	95% CI: Lower Upper p-value Test								
Int2 vs Comp	95% CI: Lower Upper p-value Test								

	Description as stated in report/paper	
102. **N missing participants and reasons** (e.g. lost to follow-up total)		
103. **N participants moved from other group and reasons**		
104. **Any other/additional results reported?**	999<br>Kaplan- Meier Curve ☐<br>Table ☐	
105. **Statistical methods used and appropriateness of these methods** *(e.g. adjust. for correlation)*		
106. **Notes**		

Response Rate

	Description as stated in report/paper					
107. **Results**	**Comparison**		**Intervention 1**		**Intervention 2**	
Note whether:<br>◯ *post-intervention OR*<br>◯ *change from baseline*<br>◯ *Unknown*<br>*And whether*<br>◯ *Adjusted OR*<br>◯ *Unadjusted*<br>◯ *Unknown*	N events	N participants	N events	N participants	N events	N participants
108. **Response rate** *(%; 95% CI)(%; 95% CI)*		Lower<br>Upper		Lower<br>Upper		Lower<br>Upper
	p-value	statistical test				
109. **N missing participants and reasons** (e.g. lost to follow-up total)						
110. **N participants moved from other group and reasons**						

	Description as stated in report/paper
111. **Any other/additional results reported?**	999 partial response ☐ overall response ☐<br>objective response ☐ stable disease ☐ Other ☐ None ☐
112. **Statistical methods used and appropriateness of these methods** *(e.g. adjustment for correlation)*	
113. **Notes:**	

Quality of Life

	Description as stated in report/paper								
114. **Results**	**Comparison**			**Intervention 1**			**Intervention 2**		
Note whether:	Median	Range	N	Median	Range	N	Median	Range	N
◯ *post-intervention OR* ◯ *change from baseline* ◯ *Unknown*<br>*And whether* ◯ *Adjusted OR* ◯ *Unadjusted* ◯ *Unknown*		Min<br>Max<br>**95% CI**:<br>Lower<br>Upper<br>**IQR**:<br>Lower<br>Upper			Min<br>Max<br>**95% CI**:<br>Lower<br>Upper<br>**IQR**:<br>Lower<br>Upper			Min<br>Max<br>**95% CI**:<br>Lower<br>Upper<br>**IQR**:<br>Lower<br>Upper	
115. **QoL results description**									
116. **N missing participants and reasons** (e.g. lost to follow-up total)									
117. **N participants moved from other group and reasons**									
118. **Any other/additional results reported?**	999								

	Description as stated in report/paper
119. **Statistical methods used and appropriateness of these methods** *(e.g. adjust. for correlation)*	
120. **Notes**	

10. Applicability

121. **Have important populations been excluded from the study?** *(consider disadvantaged populations, and possible differences in the intervention effect)*	*Yes/No/Unclear*<br>Unclear	
122. **Is the intervention likely to be aimed at disadvantaged groups?** *(e.g. lower socioeconomic groups)*	*Yes/No/Unclear*<br>Unclear	
123. **Does the study directly address the review question?** *(any issues of partial or indirect applicability)*	*Yes/No/Unclear*<br>Unclear	
124. **Notes:**		

11. Other information

	Description as stated in report/paper
125. **Key conclusions of study authors**	
126. **References to other relevant studies**	
127. **Notes:**	
