# Supplementary material for: Systematic review and network meta-analysis of the efficacy of existing treatments for patients with recurrent glioblastoma
Source: Neurooncol Adv. 2021 Apr 9;3(1):vdab052. doi: 10.1093/noajnl/vdab052 (PMC8174573; doi:10.1093/noajnl/vdab052)
Supplement: vdab052_suppl_Supplementary_Materials [file vdab052_suppl_supplementary_materials.zip › Supplementary_Material_S4.docx]

| **Study** | **Trial ID** | **Phase** | **Recurrence** | **% Male** | **Mean age** | **Activity status_a_** | **Study years** | **N** | **Intervention** | **Median OS [95% CI]** | **Median PFS [95% CI]** |
| --- | --- | --- | --- | --- | --- | --- | --- | --- | --- | --- | --- |
| Taphoorn et al.^1^, Wick et al. ^2^_b_ | NCT01290939 | III | first progression after chemoradiation | 60.6 | 54.7 | NA | 4.0 | 149 | Lomustine (CCNU) | 8.6  [7.6; 10.4] | 1.5  [1.5; 2.5] |
|  |  |  |  |  |  |  |  | 288 | Bevacizumab + Lomustine (BV+CCNU) | 9.1  [8.1; 10.1] | 4.2  [3.7; 4.3] |
| Reardon et al.^3^ _b_ | NCT02017717 | III | 100% first recurrence | NA | NA | NA | 3.0 | 165 | Bevacizumab (BV) | 10  [NA; NA] | 3.5  [NA; NA] |
|  |  |  |  |  |  |  |  | 182 | Nivolumab (NIVO) | 9.8  [NA; NA] | 1.5  [NA; NA] |
| Batchelor et al.^4^ _b_ | NCT00777153 |  | recurrent glioblastoma | 64.9 | 53.3 | 90-100  (KPS) | 7.9 | 65 | Lomustine (CCNU) | 9.8  [7.8; NA]_d_ | 2.7  [1.5; 4.1]_d_ |
|  |  | III |  |  |  |  |  | 131 | Cediranib (CDR) | 8  [6.2; 10.1]_d_ | 3.1  [2.7; 3.6]_d_ |
|  |  |  |  |  |  |  |  | 129 | Cediranib + Lomustine (CDR + CCNU) | 9.4  [7.8; 10.8]_d_ | 4.2  [3.2; 4.7]_d_ |
| Wick et al. ^5^ _b_ | NCT00295815 | III | 75.1% first recurrence 24.9% second recurrence | 64.7 | NA | 90 (KPS median) | 1.6 | 92 | Lomustine (CCNU) | 7.1  [6; 8.8] | 1.6  [1.5; 2.8] |
|  |  |  |  |  |  |  |  | 174 | Enzastaurin (ENZA) | 6.6  [5.2; 7.8] | 1.5  [1.4; 2.1] |
| Tsien et al.^6^ _b,c_ | NA | II | recurrent glioblastoma | NA | NA | NA | NA | 84 | Bevacizumab (BV) | 9.7  [NA; NA] | NA  [NA; NA] |
|  |  |  |  |  |  |  |  | 86 | Bevacizumab + Radiation Therapy (BV+radiation) | 10.1  [NA; NA] | NA  [NA; NA] |
| Friedman et al.^7^ _b_ | NCT00345163 | II | 80.8 % first recurrence 19.2% second recurrence | 68.7 | 53.1 | 70-80 (KPS) | 1.4 | 85 | Bevacizumab (BV) | 9.2  [8.2; 10.7] | 4.2  [2.9; 5.9] |
|  |  |  |  |  |  |  |  | 82 | Bevacizumab + Irinotecan (BV+CPT-11) | 8.7  [7.8; 10.9] | 5.6  [4.4; 6.2] |
| **Study** | **Trial ID** | **Phase** | **Recurrence** | **% Male** | **Mean age** | **Activity status_a_** | **Study years** | **N** | **Intervention** | **Median OS [95% CI]** | **Median PFS [95% CI]** |
| Brandes et al.^8^ _b_ | NCT01582269 | II | relapsed intracranial glioblastoma | 64.6 | 57.1 | 1 (ECOG) | 2.2 | 40 | Lomustine (CCNU) | 7.1  [5.2; 9] | 1.9  [1.7; 1.9] |
|  |  |  |  |  |  |  |  | 79 | Galunisertib + Lomustine (GAL+CCNU) | 7.2  [5.7; 9.4] | 1.8  [1.7; 1.8] |
|  |  |  |  |  |  |  |  | 39 | Galunisertib (GAL) | 6.8  [4.2; 10.8] | 1.8  [1.6; 3] |
| Taal et al.^9^, Dirven et al.^10^ _b_ | NTR1929 | II | 100% first recurrence | 62.9 | 54.7 | 1 (WHO) | 3.2 | 50 | Bevacizumab (BV) | 8  [6; 9] | 3  [3; 4] |
|  |  |  |  |  |  |  |  | 46 | Lomustine (CCNU) | 8  [6; 11] | 1  [1; 3] |
|  |  |  |  |  |  |  |  | 44 | Bevacizumab + Lomustine (BV+CCNU) | 11  [8; 12] | 4  [3; 8] |
| Cloughesy et al.^11^ _b_ | NCT01632228 | II | 100% first recurrence | 64.3 | NA | 70-80 (KPS) | 3.6 | 65 | Bevacizumab (BV) | 12.6  [7.8; NA]_d_ | 2.9  [2.8; 5.4]_d_ |
|  |  |  |  |  |  |  |  | 64 | Bevacizumab + Onartuzumab (BV+Onar) | 8.8  [7.1; 11.6]_d_ | 3.9  [2.8; 5.5]_d_ |
| Brandes et al.^12^ _b_ | NCT01860638 | II | recurrent glioblastoma | 72 | 54 | 90 (KPS median) | 3.4 | 62 | Lomustine (CCNU) | 5.5  [3.9; 7.2] | 1.8  [1.4; 2.1] |
|  |  |  |  |  |  |  |  | 61 | Lomustine + Bevacizumab (CCNU + BV) | 6.4  [5.1; 8.1] | 2.3  [1.9; 2.7] |
| Field et al.^13^ _b_ | ACTRN12610000915055 | II | 65.6% first recurrence 32.8% second recurrence | 55 | 54.3 | 70-80 (KPS) | 4.1 | 62 | Bevacizumab (BV) | 7.5  [6; 9.4]_d_ | 3.5  [1.9; 3.7] |
|  |  |  |  |  |  |  |  | 60 | Bevacizumab + Carboplatin (BV+CBP) | 6.9  [5.3; 8.2]_d_ | 3.5  [2.2; 3.7] |
|  |  |  |  |  |  |  |  |  |  |  |  |
| **Study** | **Trial ID** | **Phase** | **Recurrence** | **% Male** | **Mean age** | **Activity status_a_** | **Study years** | **N** | **Intervention** | **Median OS [95% CI]** | **Median PFS [95% CI]** |
| Galanis et al.^14,15^ _b_ | NCT00892177 | II | recurrent glioblastoma | 63.6 | 54.3 | NA | 5.1 | 38 | Bevacizumab (BV) | 7.9  [6.6; 11.3] | 3.2  [2.8; 4.6] |
|  |  |  |  |  |  |  |  | 83 | Bevacizumab + Dasatinib (BV+DST) | 7.3  [6.2; 8.7] | 3.2  [2.8; 4.6] |
| Lombardi et al. ^16,17^ _b_ | NCT02926222 | II | 100% first recurrence | 70.6 | 56.5 | 1 (ECOG) | 1.7 | 60 | Lomustine (CCNU) | 5.6  [4.7; 7.3] | 1.9  [1.8; 2.1] |
|  |  |  |  |  |  |  |  | 59 | Regorafenib (Rego) | 7.4  [5.8; 12] | 2  [1.9; 3.6] |
| Gilbert et al.^18^ _b_ | NCT00433381 | II | recurrent or progressive glioblastoma multiforme or gliosarcoma | 58.1 | 54.2 | 70-80 (KPS) | 2.8 | 57 | Bevacizumab + Irinotecan (BV+CPT-11) | 7.7  [6.7; 9.1] | 4.1  [3.4; 6.1] |
|  |  |  |  |  |  |  |  | 60 | Bevacizumab + Temozolomide (BV+TMZ) | 9.4  [6.7; 10.7] | 4.7  [3.5; 6.3] |
| Galanis et al.^19^ _b_ | NCT01648348 | II | tumor progression | 71.3 | 56.2 | NA | 3.5 | 49 | Bevacizumab (BV) | 7.4  [6.5; 12.7] | 3.2  [2.6; 4.6] |
|  |  |  |  |  |  |  |  | 52 | Bevacizumab + TRC105 (BV+TRC105) | 9.7  [6.7; 11.5] | 2.9  [2.8; 4.9] |
| Brandes et al.^20^ _b_ | NCT01474239 | II | recurrent glioblastoma multiforme (Grade IV) | 68 | 56.3 | 1 (ECOG) | 2.1 | 59 | Bevacizumab (BV) | 7.3  [5.8; 9.2] | 3.4  [3.1; 4.4] |
|  |  |  |  |  |  |  |  | 32 | Fotemustine (FTM) | 8.7  [6.3; 15.4] | 3.5  [1.9; 3.8] |
|  |  |  |  |  |  |  |  |  |  |  |  |
| **Study** | **Trial ID** | **Phase** | **Recurrence** | **% Male** | **Mean age** | **Activity status_a_** | **Study years** | **N** | **Intervention** | **Median OS [95% CI]** | **Median PFS [95% CI]** |
| Bloch et al.^21^ _b_ | NCT01814813 | II | First or second recurrence | 72.2 | NA | NA | 3.9 | 31 | Bevacizumab (BV) | 10  [8.8; 17.2] | 5.3  [3.7; 8] |
|  |  |  |  |  |  |  |  | 30 | HSPPC-96 + Bevacizumab at progression (HSPPC-96 + BV at progression) | 9.2  [5.7; 11.6] | 2.5  [2; 3.5] |
|  |  |  |  |  |  |  |  | 29 | HSPPC-96 + concomitant Bevacizumab (HSPPC-96 + concomitant BV) | 6.6  [5.4; 10.4] | 5.3  [3.7; 8] |
| Puduvalli et al.^22^ _b_ | NCT01266031 | II | recurrent glioblastoma | 65.6 | 55 | NA | 3.9 | 38 | Bevacizumab (BV) | 9.2  [5.9; 11.4] | 4.1  [1.9; 5.9] |
|  |  |  |  |  |  |  |  | 47 | Bevacizumab + Vorinostat (BV+VRS) | 7.8  [5; 9.1] | 3.7  [2.8; 4.6] |
| Weathers et al.^23^ _b_ | 2009-012186-63 | II | 69% first recurrence 31% second recurrence | 32.4 | NA | 90-100 (KPS) | 5.1 | 36 | Bevacizumab (BV) | 8.3  [6.4; 11.6] | 4.1  [2.7; 5.5] |
|  |  |  |  |  |  |  |  | 33 | Bevacizumab + Lomustine (BV+CCNU) | 9.6  [6.3; 16.7] | 4.3  [3; 8.3] |
| Hovey et al.^24^ _b_ | ACTRN12610000915055 | II | 64.6 % first recurrence 33.3 % second recurrence | 56.3 | 52.1 | 2 (ECOG) | 1.3 | 25 | Bevacizumab ceased (BV ceased) | 3  [2.4; 4.3]_d_ | 2  [NA; NA] |
|  |  |  |  |  |  |  |  | 23 | Bevacizumab (BV) | 3.4  [2.5; 5.6]_d_ | 1.8  [NA; NA] |
| Brown et al.^25^ _b_ | NCT01310855 | II | Recurrent or progressive disease | 71.1 | 53.8 | 90 (KPS median) | 2.0 | 19 | Cediranib (CDR) | 5.5  [5; 8.4]_d_ | 2.8  [2.1; 4.3]_d_ |
|  |  |  |  |  |  |  |  | 19 | Cediranib + Gefitinib (CDR+GFT) | 7.2  [5.6; 10.3]_d_ | 3.6  [2.8; 5.5]_d_ |
| **Study** | **Trial ID** | **Phase** | **Recurrence** | **% Male** | **Mean age** | **Activity status_a_** | **Study years** | **N** | **Intervention** | **Median OS [95% CI]** | **Median PFS [95% CI]** |
| Haslund et al.^26^ _b_ | 2013-003045-42 | II | recurrent glioblastoma multiforme | NA | NA | NA | NA | 10 | Bevacizumab + Irinotecan (BV+CPT-11) | 6.8  [NA; NA] | 5.4  [NA; NA] |
|  |  |  |  |  |  |  |  | 15 | Alecsat (ALE) | 5  [NA; NA] | 1  [NA; NA] |
| Reardon et al.^27^ _b_ | NA | II | 17% first recurrence 43% second recurrence 35% third recurrence 3% > 3 recurrence | 78 | NA | 90-100 (KPS) | 1.4 | 10 | Bevacizumab + Temozolomide (BV+TMZ) | 2.9  [1.1; 5.4] | 0.9  [0.7; 1.8] |
|  |  |  |  |  |  |  |  | 13 | Bevacizumab + Etoposide (BV+EPS) | 4.4  [2.5; 5.9] | 1.9  [0.9; 2.8] |
| Kunwar et al.^28^ | NCT00076986 | III | 100% first recurrence | 67 | 54.7 | 90 (KPS median) | 3.1 | 93 | Gliadel wafers | 8.8  [7.5; 11.8] | NA  [NA; NA] |
|  |  |  |  |  |  |  |  | 183 | Cintredekin besudotox (CB) | 9.1  [8.5; 11.4] | NA  [NA; NA] |
| Van Den Bent et al.^29,30^ | NCT02343406 | II | 100% first recurrence | NA | NA | 1 (WHO) | 3.7 | 86 | Lomustine or Temozolomide (CCNU/TMZ) | 8.2  [5.9; 9.5] | 1.9  [1.8; 2] |
|  |  |  |  |  |  |  |  | 86 | Depatux-M | 7.9  [6.1; 8.7] | 1.9  [1.9; 2.2] |
|  |  |  |  |  |  |  |  | 88 | Depatux-M + Temozolomide (Depatux-M+TMZ) | 9.6  [7.4; 11.8] | 2.7  [2; 3.8] |
| Dresemann et al.^31^ | NCT00154375 | III | recurrent glioblastoma multiforme | 63 | 51 | 1 (ECOG) | 3.8 | 120 | Hydroxyurea (HU) | 4.4  [3.8; 6.1]_d_ | 6  [6; 7] |
|  |  |  |  |  |  |  |  | 120 | Hydroxyurea + Imatinib (Hu+IMA) | 4.8  [4.5; 6.3]_d_ | 6  [6; 7] |
| **Study** | **Trial ID** | **Phase** | **Recurrence** | **% Male** | **Mean age** | **Activity status_a_** | **Study years** | **N** | **Intervention** | **Median OS [95% CI]** | **Median PFS [95% CI]** |
| Stupp et al.^32^, Kanner et al.^33^ | NCT00379470 | III | 11.8% first recurrence 47.3% second recurrence 40.9% third recurrence | 69.6 | 52.9 | NA | 3.2 | 117 | Best supportive care (BSC) | 6  [5.1; 7.4]_d_ | 2.1  [2.1; 3]_d_ |
|  |  |  |  |  |  |  |  | 120 | NovoTTF-100A (NovoTTF) | 6.6  [5.9; 8.1]_d_ | 2.2  [2.1; 2.7]_d_ |
| Yung et al.^34^ | NA | II | 100% first recurrence | 66 | 49.7 | 80 (KPS median) | 2.8 | 113 | Procarbazine (PCB) | NA  [NA; NA] | 1.9  [NA; NA] |
|  |  |  |  |  |  |  |  | 112 | Temozolomide (TMZ) | NA  [NA; NA] | 2.8  [NA; NA] |
| Prados et al.^35^ | NA | II | recurrent glioblastoma multiforme or anaplastic glioma | 59 | 49.7 | 80 (KPS median) | 1.8 | 60 | Carboplatin (CBP) | 4.6  [3.4; 7.2] | 1.8  [1.7; 2.9] |
|  |  |  |  |  |  |  |  | 61 | Carboplatin +RMP-7 (CBP+RMP7) | 6.2  [4.9; 8.6] | 2.2  [1.9; 2.9] |
| Van Den Bent et al.^36^ | NCT00086879 | II | Recurrent disease | 65.5 | 50.8 | 90-100 (KPS) | 1.8 | 52 | Temozolomide or Carmustine (TMZ/BCNU) | 7.3  [NA; NA] | 2.4  [NA; NA] |
|  |  |  |  |  |  |  |  | 54 | Erlotinib (ERLO) | 7.7  [NA; NA] | 1.8  [NA; NA] |
| Jaeckle et al.^37^ | NCT00329719 | II | recurrent glioblastoma | 69.9 | 53 | NA | 6.8 | 46 | Sorafenib + Temsirolimus (SORA+TMS) | 6.5  [4.2; 11.6] | NA  [NA; NA] |
|  |  |  |  |  |  |  |  | 7 | Sorafenib + Temsirolimus + Surgery (SORA+TMS+Surgery) | 6.7  [2.6; NA] | NA  [NA; NA] |
|  |  |  |  |  |  |  |  | 44 | Sorafenib + Temsirolimus (prior anti-VEGF) (SORA+TMS prior anit-VEGF) | 3.9  [3; 5.6] | NA  [NA; NA] |
|  |  |  |  |  |  |  |  |  |  |  |  |
| **Study** | **Trial ID** | **Phase** | **Recurrence** | **% Male** | **Mean age** | **Activity status_a_** | **Study years** | **N** | **Intervention** | **Median OS [95% CI]** | **Median PFS [95% CI]** |
| Bogdahn et al.^38^ | NCT00431561 | II | recurrent/ refractory AA or glioblastoma multiforme | 65.3 | NA | 90-100 (KPS) | 5.9 | 33 | Temozolomide or Procarbazine/Lomustine/Vincristine (TMZ/PCV) | 10  [7; 13] | NA  [NA; NA] |
|  |  |  |  |  |  |  |  | 28 | Trabedersen 10mM (TBD10) | 7.3  [5; 12] | NA  [NA; NA] |
|  |  |  |  |  |  |  |  | 34 | Trabedersen 80mM (TBD80) | 10.9  [5.6; 13.9] | NA  [NA; NA] |
| Narita et al.^39^, Arakawa et al.^40^ | UMIN000006970 | III | recurrent glioblastoma | 63.6 | 51.9 | < 80 (KPS) | 4.9 | 30 | Best supportive care (BSC) | 8  [4.8; 12.9] | NA  [NA; NA] |
|  |  |  |  |  |  |  |  | 58 | Best supportive care + Personalised peptide vaccination (BSC+PPV) | 8.4  [6.6; 10.6] | NA  [NA; NA] |
| Reardon et al.^41,42^ | NCT02337491 | II | First or second recurrence | 67.5 | 51.1 | NA | 3.8 | 30 | Pembrolizumab (Pembro) | 10.3  [8.5; 12.5] | 1.4  [1.4; 2.7] |
|  |  |  |  |  |  |  |  | 50 | Pembrolizumab + Bevacizumab (Pembro+BV) | 8.8  [7.7; 14.2] | 4.1  [2.8; 5.5] |
| Duerinck et al.^43,44^ | NCT01562197 | II | both "de novo" and "secondary" glioblastoma | 64.5 | 54.5 | 1 (WHO_PS) | 4.6 | 50 | Axitinib | 6.7  [4.6; 8.7] | 2.9  [2.5; 3] |
|  |  |  |  |  |  |  |  | 29 | Axitinib + Lomustine | 6.3  [4.1; 8.3] | 3  [1.4; 4.6] |
|  |  |  |  |  |  |  |  |  |  |  |  |
| **Study** | **Trial ID** | **Phase** | **Recurrence** | **% Male** | **Mean age** | **Activity status_a_** | **Study years** | **N** | **Intervention** | **Median OS [95% CI]** | **Median PFS [95% CI]** |
| Schiff et al.^45^ | NCT00562419 | II | 63.6% first recurrence 36.4% second or third recurrence | 65.2 | NA | 90-100 (KPS) | 2.9 | 27 | CT-322 2mg/kg (CT-322 2) | NA  [NA; NA] | 1.8  [NA; NA] |
|  |  |  |  |  |  |  |  | 16 | CT-322 2mg/kg+ Irinotecan (CT-322 2 +CPT-11) | NA  [NA; NA] | 4.1  [NA; NA] |
|  |  |  |  |  |  |  |  | 14 | CT-322 1mg/kg (CT-322 1) | NA  [NA; NA] | 1.4  [NA; NA] |
|  |  |  |  |  |  |  |  | 7 | CT-322 1mg/kg + Irinotecan (CT-322 1+CPT-11) | NA  [NA; NA] | 14.0  [NA; NA] |
| Sloan et al.^46^ | NCT00980343 | II | progressive or recurrent glioblastoma | 47.5 | 58.5 | NA | 2.2 | 20 | Surgery | 7.6  [5; 13.1] | NA  [NA; NA] |
|  |  |  |  |  |  |  |  | 20 | Vismodegib + Surgery (VMD+surgery) | 7.8  [3.7; 10.2] | NA  [NA; NA] |
| Cloughesy et al.^47^ | NCT02852655 | I | first or second recurrence | 38 | 57.4 | 83 (KPS mean) | 1.5 | 16 | Pembrolizumab adjuvant (Prembo adjuvant) | 7.5  [5.8; NA]_d_ | 2.4  [2.1; 2.8]_d_ |
|  |  |  |  |  |  |  |  | 16 | Pembrolizumab neoadjuvant (Prembo neoadjuvant) | 13.7  [9.5; NA]_d_ | 3.3  [2.4; 12.5]_d_ |
| Short et al.^48^, Twelves et al. ^49^ | NCT01812616 | II | 100% first recurrence | 61.9 | 58 | 90 (KPS median) | 4.8 | 9 | Temozolomide (TMZ) | 12.3  [NA; NA] | NA  [NA; NA] |
|  |  |  |  |  |  |  |  | 12 | Temozolomide + Cannabidol + delta-9-tetrahydrocannabiol) (TMZ+CBD+THC) | 18.3  [NA; NA] | NA  [NA; NA] |
| Sun et al.^50^ | NCT00335075 | III | tumor progression or recurrence | NA | NA | NA | 0.9 | NA | Temozolomide (TMZ) | NA  [NA; NA] | NA  [NA; NA] |
|  |  |  |  |  |  |  |  | NA | Semustine (SMT) | NA  [NA; NA] | NA  [NA; NA] |

_a_ Reported activity status of patients (KPS, ECOG, WHO); Either median or most reported category.

_b_ Study included in NMA

_c_ included only for OS

_d_ Reconstruction of 95% CI

**1.** Taphoorn M, Bottomley A, Coens C, et al. Health-related quality of life (HRQoL) in patients with progressive glioblastoma treated with combined bevacizumab and lomustine versus lomustine only (randomized phase iii EORTC study 26101). *Neuro-Oncology.* 2016; 18:vi157.

**2.** Wick W, Gorlia T, Bendszus M, et al. Lomustine and bevacizumab in progressive glioblastoma. 2017; 377(20):1954-1963.

**3.** Reardon DA, Omuro A, Brandes AA, et al. Randomized phase 3 study evaluating the efficacy and safety of nivolumab vs bevacizumab in patients with recurrent glioblastoma: Checkmate 143. *Neuro-Oncology.* 2017; 19:iii21.

**4.** Batchelor TT, Mulholland P, Neyns B, et al. Phase III randomized trial comparing the efficacy of cediranib as monotherapy, and in combination with lomustine, versus lomustine alone in patients with recurrent glioblastoma. *J Clin Oncol.* 2013; 31(26):3212-3218.

**5.** Wick W, Puduvalli VK, Chamberlain MC, et al. Phase III study of enzastaurin compared with lomustine in the treatment of recurrent intracranial glioblastoma. *J Clin Oncol.* 2010; 28(7):1168-1174.

**6.** Tsien C, Pugh S, Dicker A, et al. Randomized Phase II Trial of Re-Irradiation and Concurrent Bevacizumab versus Bevacizumab Alone as Treatment for Recurrent Glioblastoma (NRG Oncology/RTOG 1205): Initial Outcomes and RT Plan Quality Report. 2019; 105(1):S78.

**7.** Friedman HS, Prados MD, Wen PY, et al. Bevacizumab alone and in combination with irinotecan in recurrent glioblastoma. *J Clin Oncol.* 2009; 27(28):4733-4740.

**8.** Brandes AA, Carpentier AF, Kesari S, et al. A Phase II randomized study of galunisertib monotherapy or galunisertib plus lomustine compared with lomustine monotherapy in patients with recurrent glioblastoma. *Neuro Oncol.* 2016; 18(8):1146-1156.

**9.** Taal W, Oosterkamp HM, Walenkamp AM, et al. Single-agent bevacizumab or lomustine versus a combination of bevacizumab plus lomustine in patients with recurrent glioblastoma (BELOB trial): a randomised controlled phase 2 trial. *Lancet Oncol.* 2014; 15(9):943-953.

**10.** Dirven L, van den Bent MJ, Bottomley A, et al. The impact of bevacizumab on health-related quality of life in patients treated for recurrent glioblastoma: results of the randomised controlled phase 2 BELOB trial. *Eur J Cancer.* 2015; 51(10):1321-1330.

**11.** Cloughesy T, Finocchiaro G, Belda-Iniesta C, et al. Randomized, double-blind, placebo-controlled, multicenter phase II study of onartuzumab plus bevacizumab versus placebo plus bevacizumab in patients with recurrent glioblastoma: Efficacy, safety, and hepatocyte growth factor and O6-methylguanine-DNA methyltransferase biomarker analyses. *Journal of Clinical Oncology.* 2017; 35(3):343-351.

**12.** Brandes AA, Gil-Gil M, Saran F, et al. A Randomized Phase II Trial (TAMIGA) Evaluating the Efficacy and Safety of Continuous Bevacizumab Through Multiple Lines of Treatment for Recurrent Glioblastoma. *Oncologist.* 2019; 24(4):521-528.

**13.** Field KM, King MT, Simes J, et al. Health-related quality of life outcomes from CABARET: a randomized phase 2 trial of carboplatin and bevacizumab in recurrent glioblastoma. 2017; 133(3):623-631.

**14.** Galanis E, Anderson SK, Anastasiadis P, et al. NCCTG N0872 (Alliance): A randomized placebo-controlled phase II trial of bevacizumab plus dasatinib in patients with recurrent glioblastoma (GBM): American Society of Clinical Oncology; 2015.

**15.** Galanis E, Anderson SK, Twohy EL, et al. A phase 1 and randomized, placebo‐controlled phase 2 trial of bevacizumab plus dasatinib in patients with recurrent glioblastoma: Alliance/North Central Cancer Treatment Group N0872. 2019; 125(21):3790-3800.

**16.** Lombardi G, De Salvo GL, Brandes AA, et al. Regorafenib compared with lomustine in patients with relapsed glioblastoma (REGOMA): a multicentre, open-label, randomised, controlled, phase 2 trial. *The Lancet Oncology.* 2019; 20(1):110-119.

**17.** Lombardi G, De Salvo GL, Brandes AA, et al. REGOMA: A randomized, multicenter, controlled open-label phase II clinical trial evaluating regorafenib activity in relapsed glioblastoma patients. *Annals of Oncology.* 2017; 28:v610.

**18.** Gilbert MR, Pugh SL, Aldape K, et al. NRG oncology RTOG 0625: a randomized phase II trial of bevacizumab with either irinotecan or dose-dense temozolomide in recurrent glioblastoma. *J Neurooncol.* 2017; 131(1):193-199.

**19.** Galanis E, Anderson SK, Butowski NA, et al. NCCTG N1174: Phase I/comparative randomized phase (Ph) II trial of TRC105 plus bevacizumab versus bevacizumab in recurrent glioblastoma (GBM)(Alliance): American Society of Clinical Oncology; 2017.

**20.** Brandes AA, Finocchiaro G, Zagonel V, et al. AVAREG: a phase II, randomized, noncomparative study of fotemustine or bevacizumab for patients with recurrent glioblastoma. *Neuro Oncol.* 2016; 18(9):1304-1312.

**21.** Bloch O, Shi Q, Anderson SK, et al. Alliance a071101: A phase II randomized trial comparing the efficacy of heat shock protein peptide complex-96 (HSPPC-96) vaccine given with bevacizumab versus bevacizumab alone in the treatment of surgically resectable recurrent glioblastoma. *Neuro-Oncology.* 2017; 19:vi29.

**22.** Puduvalli VK, Wu J, Yuan Y, et al. Brain Tumor Trials Collaborative Bayesian Adaptive Randomized Phase II trial of bevacizumab plus vorinostat versus bevacizumab alone in adults with recurrent glioblastoma (BTTC-1102): American Society of Clinical Oncology; 2015.

**23.** Weathers SP, Han X, Liu DD, et al. A randomized phase II trial of standard dose bevacizumab versus low dose bevacizumab plus lomustine (CCNU) in adults with recurrent glioblastoma. *J Neurooncol.* 2016; 129(3):487-494.

**24.** Hovey EJ, Field KM, Rosenthal MA, et al. Continuing or ceasing bevacizumab beyond progression in recurrent glioblastoma: An exploratory randomized phase II trial. *Neuro-Oncology Practice.* 2017; 4(3):171-181.

**25.** Brown N, McBain C, Nash S, et al. Multi-Center Randomized Phase II Study Comparing Cediranib plus Gefitinib with Cediranib plus Placebo in Subjects with Recurrent/Progressive Glioblastoma. *PLoS One.* 2016; 11(5):e0156369.

**26.** Haslund C, Muhic A, Lukacova S, et al. An open-labelled, randomized phase II study in patients with recurrent glioblastoma multiforme comparing progression free survival of alecsat (autologous lymphoid effector cells specific against tumour-cells) versus bevacizumab/irinotecan. *Neuro-Oncology.* 2016; 18:iv6.

**27.** Reardon DA, Desjardins A, Peters K, et al. Phase II study of metronomic chemotherapy with bevacizumab for recurrent glioblastoma after progression on bevacizumab therapy. *J Neurooncol.* 2011; 103(2):371-379.

**28.** Kunwar S, Chang S, Westphal M, et al. Phase III randomized trial of CED of IL13-PE38QQR vs Gliadel wafers for recurrent glioblastoma. *Neuro Oncol.* 2010; 12(8):871-881.

**29.** Van Den Bent M, Eoli M, Sepulveda JM, et al. First results of the randomized phase ii study on depatux-m alone, depatux-m in combination with temozolomide and either temozolomide or lomustine in recurrent EGFR amplified glioblastoma: First report from intellance 2/eortc trial 1410. *Neuro-Oncology.* 2017; 19:vi316.

**30.** Van den Bent M, Eoli M, Sepulveda JM, et al. INTELLANCE 2/EORTC 1410 randomized phase II study of Depatux-M alone and with temozolomide vs temozolomide or lomustine in recurrent EGFRamplified glioblastoma. 2019.

**31.** Dresemann G, Weller M, Rosenthal MA, et al. Imatinib in combination with hydroxyurea versus hydroxyurea alone as oral therapy in patients with progressive pretreated glioblastoma resistant to standard dose temozolomide. *J Neurooncol.* 2010; 96(3):393-402.

**32.** Stupp R, Wong ET, Kanner AA, et al. NovoTTF-100A versus physician's choice chemotherapy in recurrent glioblastoma: a randomised phase III trial of a novel treatment modality. *Eur J Cancer.* 2012; 48(14):2192-2202.

**33.** Kanner AA, Wong ET, Villano JL, Ram Z. Post Hoc analyses of intention-to-treat population in phase III comparison of NovoTTF-100A system versus best physician's choice chemotherapy. *Semin Oncol.* 2014; 41 Suppl 6:S25-34.

**34.** Yung WA, Albright R, Olson J, et al. A phase II study of temozolomide vs. procarbazine in patients with glioblastoma multiforme at first relapse. *British Journal of Cancer.* 2000; 83(5):588.

**35.** Prados MD, Schold SC, Jr., Fine HA, et al. A randomized, double-blind, placebo-controlled, phase 2 study of RMP-7 in combination with carboplatin administered intravenously for the treatment of recurrent malignant glioma. *Neuro Oncol.* 2003; 5(2):96-103.

**36.** Van den Bent MJ, Brandes AA, Rampling R, et al. Randomized phase II trial of erlotinib versus temozolomide or carmustine in recurrent glioblastoma: EORTC brain tumor group study 26034. *J Clin Oncol.* 2009; 27(8):1268-1274.

**37.** Jaeckle KA, Schiff D, Anderson SK, et al. NCCTG (Alliance) N0572: A phase II trial of sorafenib and temsirolimus in recurrent glioblastoma (GBM) patients who progress following prior RT/temozolomide (TMZ) and VEGF inhibitors (VEGFi): American Society of Clinical Oncology; 2014.

**38.** Bogdahn U, Hau P, Stockhammer G, et al. Targeted therapy for high-grade glioma with the TGF-β2 inhibitor trabedersen: results of a randomized and controlled phase IIb study. *Neuro-Oncology.* 2010; 13(1):132-142.

**39.** Narita Y, Arakawa Y, Yamasaki F, et al. A randomized, double-blind, phase III trial of personalized peptide vaccination for recurrent glioblastoma. *Neuro-Oncology.* 2019; 21(3):348-359.

**40.** Arakawa Y, Nagane M, Hirose Y, et al. Randomized, double-blind, phase III trial of a personalized peptide vaccination for recurrent glioblastoma patients. *Cancer Science.* 2018; 109:1126.

**41.** Reardon DA, De Groot JF, Colman H, et al. Safety of pembrolizumab in combination with bevacizumab in recurrent glioblastoma (rGBM): American Society of Clinical Oncology; 2016.

**42.** Reardon DA, Nayak L, Peters KB, et al. Phase II study of pembrolizumab or pembrolizumab plus bevacizumab for recurrent glioblastoma (rGBM) patients. *Journal of Clinical Oncology.* 2018; 36(15).

**43.** Duerinck J, Du Four S, Bouttens F, et al. Randomized phase II trial comparing axitinib with the combination of axitinib and lomustine in patients with recurrent glioblastoma. *J Neurooncol.* 2018; 136(1):115-125.

**44.** Duerinck J, Du Four S, Vandervorst F, et al. Randomized phase II study of axitinib versus physicians best alternative choice of therapy in patients with recurrent glioblastoma. 2016; 128(1):147-155.

**45.** Schiff D, Kesari S, de Groot J, et al. Phase 2 study of CT-322, a targeted biologic inhibitor of VEGFR-2 based on a domain of human fibronectin, in recurrent glioblastoma. *Invest New Drugs.* 2015; 33(1):247-253.

**46.** Sloan AE, Nock CJ, Ye X, et al. Targeting glioma-initiating cells in GBM: ABTC-0904, a randomized phase 0/II study targeting the Sonic Hedgehog-signaling pathway: American Society of Clinical Oncology; 2014.

**47.** Cloughesy TF, Mochizuki AY, Orpilla JR, et al. Neoadjuvant anti-PD-1 immunotherapy promotes a survival benefit with intratumoral and systemic immune responses in recurrent glioblastoma. *Nat Med.* 2019; 25(3):477-486.

**48.** Short SC, Little C. A 2-part safety and exploratory efficacy randomised double-blind, placebo-controlled study of a 1:1 ratio of cannabidiol and delta-9-tetrahydrocannabinol (CBD: THC) plus doseintense temozolomide in patients with recurrent glioblastoma multiforme (GBM). *Neuro-Oncology.* 2017; 19:vi13.

**49.** Twelves C, Short S, Wright S. A two-part safety and exploratory efficacy randomized double-blind, placebo-controlled study of a 1:1 ratio of the cannabinoids cannabidiol and delta-9-tetrahydrocannabinol (CBD: THC) plus dose-intense temozolomide in patients with recurrent glioblastoma multiforme (GBM). *Journal of Clinical Oncology.* 2017; 35(15):2046-2046.

**50.** Sun J, Yang XJ, Yang SY. [Multicenter randomized controlled study of temozolomide versus semustine in the treatment of recurrent malignant glioma]. *Zhonghua Yi Xue Za Zhi.* 2013; 93(3):165-168.
