# Supplementary material for: Systematic review and network meta-analysis of the efficacy of existing treatments for patients with recurrent glioblastoma
Source: Neurooncol Adv. 2021 Apr 9;3(1):vdab052. doi: 10.1093/noajnl/vdab052 (PMC8174573; doi:10.1093/noajnl/vdab052)
Supplement: vdab052_suppl_Supplementary_Materials [file vdab052_suppl_supplementary_materials.zip › Supplementary_Material_S5.docx]

| Supp. 5: Hazard Ratio (HR) Matrix of overall survival (OS) | | | | |  |  |  |  |  |  |  |  |
| --- | --- | --- | --- | --- | --- | --- | --- | --- | --- | --- | --- | --- |
|  | **BV + EPS** | **BV + CCNU** | **FTM** | **BV + TMZ** | **CCNU** | **CDR + GFT** | **CCNU + GAL** | **BV + Radiation** | **CCNU + CDR** | **BV** | **BV ceased** |  |
| **Rego** | 1.31  [0.27; 6.65] | 0.87  [0.49; 1.53] | 0.88  [0.35; 2.23] | 0.79  [0.3; 2.08] | 0.78  [0.46; 1.33] | 0.8  [0.26; 2.47] | 0.76  [0.35; 1.66] | 0.75  [0.33; 1.66] | 0.73  [0.37; 1.42] | 0.71  [0.36; 1.41] | 0.71  [0.24; 2.08] |  |
| **BV + EPS** |  | 0.66  [0.14; 2.98] | 0.67  [0.14; 3.31] | 0.61  [0.17; 2.2] | 0.59  [0.13; 2.74] | 0.61  [0.1; 3.79] | 0.58  [0.11; 2.93] | 0.57  [0.13; 2.64] | 0.56  [0.11; 2.77] | 0.54  [0.13; 2.37] | 0.54  [0.1; 2.93] |  |
| **BV + CCNU** |  |  | 1.02  [0.49; 2.15] | 0.91  [0.41; 2.05] | 0.9  [0.72; 1.13] | 0.92  [0.33; 2.57] | 0.88  [0.47; 1.57] | 0.86  [0.48; 1.54] | 0.85  [0.51; 1.37] | 0.82  [0.55; 1.24] | 0.82  [0.31; 2.04] |  |
| **FTM** |  |  |  | 0.9  [0.35; 2.3] | 0.88  [0.41; 1.82] | 0.9  [0.26; 3.15] | 0.86  [0.33; 2.24] | 0.84  [0.39; 1.82] | 0.83  [0.34; 1.94] | 0.81  [0.43; 1.49] | 0.8  [0.28; 2.22] |  |
| **BV + TMZ** |  |  |  |  | 0.98  [0.44; 2.26] | 1.01  [0.28; 3.66] | 0.96  [0.35; 2.57] | 0.94  [0.42; 2.07] | 0.93  [0.36; 2.33] | 0.9  [0.46; 1.77] | 0.89  [0.31; 2.57] |  |
| **CCNU** |  |  |  |  |  | 1.03  [0.39; 2.77] | 0.98  [0.55; 1.68] | 0.96  [0.52; 1.75] | 0.95  [0.61; 1.45] | 0.92  [0.6; 1.42] | 0.91  [0.36; 2.27] |  |
| **CDR + GFT** |  |  |  |  |  |  | 0.95  [0.3; 2.99] | 0.93  [0.29; 2.97] | 0.92  [0.35; 2.43] | 0.89  [0.31; 2.61] | 0.89  [0.23; 3.41] |  |
| **CCNU + GAL** |  |  |  |  |  |  |  | 0.98  [0.43; 2.23] | 0.97  [0.48; 1.95] | 0.94  [0.47; 1.95] | 0.93  [0.32; 2.75] |  |
| **BV + Radiation** |  |  |  |  |  |  |  |  | 0.99  [0.46; 2] | 0.96  [0.63; 1.48] | 0.95  [0.37; 2.41] |  |
| **CCNU + CDR** |  |  |  |  |  |  |  |  |  | 0.97  [0.54; 1.8] | 0.96  [0.34; 2.71] |  |
| **BV** |  |  |  |  |  |  |  |  |  |  | 0.99  [0.43; 2.25] |  |
| **BV ceased** |  |  |  |  |  |  |  |  |  |  |  |  |
| **ENZA** |  |  |  |  |  |  |  |  |  |  |  |  |
| **GAL** |  |  |  |  |  |  |  |  |  |  |  |  |
| **HSPPC-96 + BV at progression** |  |  |  |  |  |  |  |  |  |  |  |  |
| **NIVO** |  |  |  |  |  |  |  |  |  |  |  |  |
| **BV+CPT-11** |  |  |  |  |  |  |  |  |  |  |  |  |
| **BV + CBP** |  |  |  |  |  |  |  |  |  |  |  |  |
| **BV + DST** |  |  |  |  |  |  |  |  |  |  |  |  |
| **CDR** |  |  |  |  |  |  |  |  |  |  |  |  |
| **BV + VRS** |  |  |  |  |  |  |  |  |  |  |  |  |
| **HSPPC-96 + concomitant BV** |  |  |  |  |  |  |  |  |  |  |  |  |
| **ALE** |  |  |  |  |  |  |  |  |  |  |  |  |
|  |  |  |  |  |  |  |  |  |  |  |  |  |
|  |  |  |  |  |  |  |  |  |  |  |  |  |
|  |  |  |  |  |  |  |  |  |  |  |  |  |
|  |  |  |  |  |  |  |  |  |  |  |  |  |
|  | **ENZA** | **GAL** | **HSPPC-96 + BV at progression** | **NIVO** | **BV+CPT-11** | **BV + CBP** | **BV + DST** | **CDR** | **BV + VRS** | **HSPPC-96 + concomitant BV** | **ALE** | **BV + Onar** |
| **Rego** | 0.72  [0.38; 1.36] | 0.71  [0.31; 1.61] | 0.69  [0.26; 1.9] | 0.7  [0.33; 1.47] | 0.67  [0.3; 1.5] | 0.66  [0.29; 1.58] | 0.64  [0.26; 1.51] | 0.61  [0.31; 1.22] | 0.58  [0.24; 1.46] | 0.53  [0.19; 1.42] | 0.43  [0.1; 1.74] | 0.49  [0.21; 1.14] |
| **BV + EPS** | 0.55  [0.11; 2.71] | 0.54  [0.11; 2.78] | 0.53  [0.1; 2.68] | 0.53  [0.12; 2.35] | 0.51  [0.13; 2.03] | 0.5  [0.11; 2.39] | 0.49  [0.1; 2.3] | 0.47  [0.09; 2.23] | 0.44  [0.09; 2.17] | 0.4  [0.08; 2.05] | 0.33  [0.05; 2.02] | 0.37  [0.08; 1.75] |
| **BV + CCNU** | 0.83  [0.53; 1.27] | 0.82  [0.41; 1.6] | 0.8  [0.34; 1.85] | 0.8  [0.49; 1.34] | 0.77  [0.42; 1.4] | 0.76  [0.4; 1.45] | 0.73  [0.37; 1.45] | 0.7  [0.43; 1.16] | 0.67  [0.32; 1.41] | 0.61  [0.27; 1.41] | 0.5  [0.13; 1.81] | 0.56  [0.3; 1.07] |
| **FTM** | 0.81  [0.35; 1.82] | 0.8  [0.3; 2.16] | 0.79  [0.3; 2.05] | 0.79  [0.39; 1.53] | 0.75  [0.34; 1.63] | 0.75  [0.34; 1.69] | 0.72  [0.3; 1.65] | 0.69  [0.29; 1.64] | 0.66  [0.27; 1.55] | 0.59  [0.23; 1.57] | 0.49  [0.12; 1.91] | 0.55  [0.24; 1.21] |
| **BV + TMZ** | 0.9  [0.37; 2.23] | 0.89  [0.31; 2.53] | 0.88  [0.32; 2.4] | 0.88  [0.41; 1.85] | 0.84  [0.5; 1.42] | 0.83  [0.35; 1.94] | 0.8  [0.33; 1.93] | 0.77  [0.3; 1.93] | 0.73  [0.29; 1.86] | 0.66  [0.24; 1.86] | 0.54  [0.15; 1.91] | 0.62  [0.26; 1.43] |
| **CCNU** | 0.92  [0.63; 1.31] | 0.91  [0.48; 1.71] | 0.89  [0.38; 2.1] | 0.89  [0.53; 1.53] | 0.86  [0.46; 1.61] | 0.85  [0.44; 1.67] | 0.82  [0.41; 1.63] | 0.79  [0.51; 1.22] | 0.75  [0.35; 1.62] | 0.68  [0.29; 1.59] | 0.56  [0.14; 2.04] | 0.63  [0.32; 1.21] |
| **CDR + GFT** | 0.9  [0.31; 2.56] | 0.89  [0.27; 2.87] | 0.87  [0.23; 3.19] | 0.87  [0.29; 2.62] | 0.83  [0.27; 2.68] | 0.83  [0.26; 2.77] | 0.8  [0.24; 2.68] | 0.77  [0.31; 1.87] | 0.73  [0.21; 2.5] | 0.66  [0.18; 2.46] | 0.54  [0.1; 2.65] | 0.61  [0.19; 1.97] |
| **CCNU + GAL** | 0.94  [0.49; 1.83] | 0.93  [0.54; 1.69] | 0.91  [0.33; 2.57] | 0.91  [0.42; 2.03] | 0.88  [0.39; 2.08] | 0.87  [0.37; 2.18] | 0.84  [0.34; 2.07] | 0.8  [0.4; 1.63] | 0.77  [0.3; 2] | 0.69  [0.25; 1.92] | 0.57  [0.13; 2.32] | 0.64  [0.27; 1.55] |
| **BV + Radiation** | 0.96  [0.48; 1.92] | 0.95  [0.39; 2.24] | 0.93  [0.4; 2.18] | 0.93  [0.55; 1.56] | 0.89  [0.49; 1.68] | 0.89  [0.46; 1.76] | 0.85  [0.42; 1.72] | 0.82  [0.38; 1.72] | 0.78  [0.36; 1.65] | 0.7  [0.3; 1.66] | 0.58  [0.15; 2.14] | 0.66  [0.34; 1.27] |
| **CCNU + CDR** | 0.98  [0.55; 1.7] | 0.96  [0.45; 2.09] | 0.95  [0.37; 2.49] | 0.95  [0.48; 1.88] | 0.91  [0.44; 2.01] | 0.9  [0.41; 2.03] | 0.87  [0.38; 1.94] | 0.83  [0.59; 1.2] | 0.79  [0.33; 1.89] | 0.72  [0.28; 1.84] | 0.59  [0.14; 2.33] | 0.67  [0.3; 1.48] |
| **BV** | 1  [0.56; 1.77] | 0.99  [0.45; 2.13] | 0.97  [0.47; 2.01] | 0.97  [0.72; 1.31] | 0.93  [0.59; 1.45] | 0.93  [0.55; 1.57] | 0.89  [0.5; 1.57] | 0.86  [0.46; 1.57] | 0.81  [0.43; 1.52] | 0.74  [0.35; 1.54] | 0.6  [0.16; 2.04] | 0.68  [0.41; 1.13] |
| **BV ceased** | 1.01  [0.37; 2.76] | 1  [0.32; 3.13] | 0.98  [0.32; 2.9] | 0.98  [0.41; 2.35] | 0.94  [0.37; 2.4] | 0.93  [0.35; 2.5] | 0.9  [0.33; 2.42] | 0.86  [0.32; 2.42] | 0.82  [0.29; 2.32] | 0.74  [0.24; 2.29] | 0.61  [0.14; 2.72] | 0.69  [0.26; 1.85] |
| **ENZA** |  | 0.99  [0.48; 2.09] | 0.97  [0.39; 2.46] | 0.97  [0.51; 1.88] | 0.93  [0.45; 1.91] | 0.92  [0.44; 1.98] | 0.89  [0.4; 1.96] | 0.85  [0.49; 1.52] | 0.81  [0.34; 1.89] | 0.73  [0.29; 1.87] | 0.6  [0.15; 2.31] | 0.68  [0.32; 1.47] |
| **GAL** |  |  | 0.98  [0.34; 2.82] | 0.98  [0.43; 2.23] | 0.94  [0.38; 2.29] | 0.93  [0.37; 2.4] | 0.9  [0.34; 2.38] | 0.86  [0.4; 1.87] | 0.82  [0.31; 2.2] | 0.74  [0.25; 2.14] | 0.61  [0.13; 2.67] | 0.69  [0.28; 1.78] |
| **HSPPC-96 + BV at progression** |  |  |  | 1  [0.46; 2.18] | 0.96  [0.41; 2.26] | 0.95  [0.39; 2.34] | 0.92  [0.36; 2.33] | 0.88  [0.34; 2.29] | 0.84  [0.32; 2.17] | 0.76  [0.35; 1.61] | 0.62  [0.15; 2.59] | 0.7  [0.29; 1.73] |
| **NIVO** |  |  |  |  | 0.96  [0.56; 1.65] | 0.95  [0.53; 1.73] | 0.92  [0.47; 1.73] | 0.88  [0.45; 1.71] | 0.84  [0.41; 1.62] | 0.76  [0.34; 1.69] | 0.62  [0.17; 2.16] | 0.7  [0.39; 1.25] |
| **BV+CPT-11** |  |  |  |  |  | 0.99  [0.5; 1.98] | 0.96  [0.47; 1.97] | 0.92  [0.43; 1.93] | 0.87  [0.4; 1.87] | 0.79  [0.33; 1.84] | 0.65  [0.19; 2.07] | 0.73  [0.37; 1.44] |
| **BV + CBP** |  |  |  |  |  |  | 0.96  [0.45; 2.05] | 0.92  [0.41; 2.04] | 0.88  [0.39; 1.95] | 0.79  [0.33; 1.92] | 0.65  [0.16; 2.47] | 0.74  [0.36; 1.55] |
| **BV + DST** |  |  |  |  |  |  |  | 0.96  [0.43; 2.2] | 0.91  [0.38; 2.08] | 0.82  [0.32; 2.16] | 0.68  [0.17; 2.56] | 0.77  [0.37; 1.65] |
| **CDR** |  |  |  |  |  |  |  |  | 0.95  [0.39; 2.28] | 0.86  [0.33; 2.21] | 0.71  [0.17; 2.81] | 0.8  [0.36; 1.8] |
| **BV + VRS** |  |  |  |  |  |  |  |  |  | 0.9  [0.34; 2.35] | 0.74  [0.18; 3.01] | 0.84  [0.38; 1.88] |
| **HSPPC-96 + concomitant BV** |  |  |  |  |  |  |  |  |  |  | 0.82  [0.19; 3.53] | 0.93  [0.38; 2.31] |
| **ALE** |  |  |  |  |  |  |  |  |  |  |  | 1.13  [0.3; 4.48] |
