# Supplementary material for: Systematic review and network meta-analysis of the efficacy of existing treatments for patients with recurrent glioblastoma
Source: Neurooncol Adv. 2021 Apr 9;3(1):vdab052. doi: 10.1093/noajnl/vdab052 (PMC8174573; doi:10.1093/noajnl/vdab052)
Supplement: vdab052_suppl_Supplementary_Materials [file vdab052_suppl_supplementary_materials.zip › Supplementary_Material_S6.docx]

|  |  |  |  |  |  |  |  |  |  |  |  |  |
| --- | --- | --- | --- | --- | --- | --- | --- | --- | --- | --- | --- | --- |
|  | Supp. 6: Hazard Ratio (HR) Matrix of progression free survival (PFS) | | | | | | |  |  |  |  |  |
|  |  | BV + TMZ | BV+CPT-11 | BV + Onar | BV ceased | BV + CCNU | HSPPC-96 + concomitant BV | FTM | BV + CBP | BV | BV + DST | BV + VRS |
|  | BV + EPS | 0.47  [0.13; 1.66] | 0.42  [0.11; 1.72] | 0.42  [0.09; 1.92] | 0.4  [0.07; 2.18] | 0.34  [0.08; 1.55] | 0.35  [0.07; 1.77] | 0.34  [0.07; 1.69] | 0.32  [0.07; 1.45] | 0.32  [0.08; 1.38] | 0.31  [0.07; 1.4] | 0.28  [0.06; 1.35] |
|  | BV + TMZ |  | 0.89  [0.52; 1.54] | 0.9  [0.39; 2.12] | 0.85  [0.29; 2.53] | 0.74  [0.33; 1.65] | 0.76  [0.27; 2.11] | 0.72  [0.28; 1.83] | 0.68  [0.29; 1.59] | 0.68  [0.35; 1.37] | 0.66  [0.27; 1.59] | 0.6  [0.24; 1.53] |
|  | BV+CPT-11 |  |  | 1  [0.51; 1.97] | 0.95  [0.37; 2.45] | 0.82  [0.45; 1.51] | 0.85  [0.36; 2.03] | 0.81  [0.38; 1.77] | 0.76  [0.39; 1.51] | 0.76  [0.49; 1.18] | 0.74  [0.36; 1.5] | 0.67  [0.31; 1.42] |
|  | BV + Onar |  |  |  | 0.95  [0.36; 2.59] | 0.82  [0.43; 1.59] | 0.85  [0.34; 2.07] | 0.81  [0.36; 1.82] | 0.76  [0.37; 1.56] | 0.76  [0.46; 1.27] | 0.74  [0.35; 1.54] | 0.67  [0.3; 1.49] |
|  | BV ceased |  |  |  |  | 0.86  [0.34; 2.24] | 0.89  [0.28; 2.71] | 0.85  [0.31; 2.4] | 0.8  [0.29; 2.08] | 0.8  [0.35; 1.84] | 0.77  [0.29; 2.1] | 0.7  [0.25; 1.93] |
|  | BV + CCNU |  |  |  |  |  | 1.03  [0.43; 2.42] | 0.98  [0.47; 2.09] | 0.92  [0.48; 1.8] | 0.93  [0.61; 1.41] | 0.89  [0.44; 1.79] | 0.81  [0.38; 1.76] |
|  | HSPPC-96 + concomitant BV |  |  |  |  |  |  | 0.95  [0.37; 2.52] | 0.9  [0.36; 2.22] | 0.9  [0.43; 1.89] | 0.87  [0.35; 2.2] | 0.79  [0.29; 2.11] |
|  | FTM |  |  |  |  |  |  |  | 0.94  [0.42; 2.06] | 0.94  [0.5; 1.74] | 0.91  [0.39; 2.06] | 0.83  [0.34; 2.02] |
|  | BV + CBP |  |  |  |  |  |  |  |  | 1  [0.6; 1.7] | 0.97  [0.44; 2.06] | 0.88  [0.39; 1.95] |
|  | BV |  |  |  |  |  |  |  |  |  | 0.97  [0.55; 1.66] | 0.88  [0.47; 1.64] |
|  | BV + DST |  |  |  |  |  |  |  |  |  |  | 0.91  [0.4; 2.13] |
|  | BV + VRS |  |  |  |  |  |  |  |  |  |  |  |
|  | CCNU + CDR |  |  |  |  |  |  |  |  |  |  |  |
|  | CDR + GFT |  |  |  |  |  |  |  |  |  |  |  |
|  | HSPPC-96 + BV at progression |  |  |  |  |  |  |  |  |  |  |  |
|  | CDR |  |  |  |  |  |  |  |  |  |  |  |
|  | Rego |  |  |  |  |  |  |  |  |  |  |  |
|  | CCNU |  |  |  |  |  |  |  |  |  |  |  |
|  | NIVO |  |  |  |  |  |  |  |  |  |  |  |
|  | CCNU + GAL |  |  |  |  |  |  |  |  |  |  |  |
|  | GAL |  |  |  |  |  |  |  |  |  |  |  |
|  | ENZA |  |  |  |  |  |  |  |  |  |  |  |
|  |  |  |  |  |  |  |  |  |  |  |  |  |
|  |  |  |  |  |  |  |  |  |  |  |  |  |
|  |  |  |  |  |  |  |  |  |  |  |  |  |
|  |  |  |  |  |  |  |  |  |  |  |  |  |
|  |  | CCNU + CDR | CDR + GFT | HSPPC-96 + BV at progression | CDR | Rego | CCNU | NIVO | CCNU + GAL | GAL | ENZA | ALE |
|  | BV + EPS | 0.21  [0.04; 0.98] | 0.19  [0.03; 1.16] | 0.16  [0.03; 0.79] | 0.15  [0.03; 0.68] | 0.14  [0.03; 0.67] | 0.14  [0.03; 0.61] | 0.13  [0.03; 0.59] | 0.13  [0.03; 0.64] | 0.12  [0.02; 0.66] | 0.12  [0.03; 0.59] | 0.07  [0.01; 0.41] |
|  | BV + TMZ | 0.44  [0.17; 1.09] | 0.41  [0.11; 1.51] | 0.34  [0.12; 0.93] | 0.32  [0.13; 0.8] | 0.3  [0.11; 0.81] | 0.29  [0.13; 0.67] | 0.29  [0.14; 0.62] | 0.27  [0.1; 0.73] | 0.27  [0.09; 0.76] | 0.27  [0.11; 0.65] | 0.15  [0.04; 0.52] |
|  | BV+CPT-11 | 0.5  [0.23; 1.04] | 0.46  [0.14; 1.5] | 0.38  [0.16; 0.9] | 0.36  [0.16; 0.74] | 0.34  [0.15; 0.75] | 0.33  [0.17; 0.61] | 0.32  [0.19; 0.55] | 0.3  [0.13; 0.69] | 0.3  [0.12; 0.72] | 0.3  [0.14; 0.62] | 0.16  [0.05; 0.52] |
|  | BV + Onar | 0.49  [0.22; 1.13] | 0.46  [0.14; 1.55] | 0.38  [0.16; 0.93] | 0.36  [0.16; 0.8] | 0.34  [0.15; 0.79] | 0.33  [0.17; 0.64] | 0.32  [0.18; 0.59] | 0.3  [0.12; 0.72] | 0.3  [0.12; 0.75] | 0.3  [0.14; 0.65] | 0.16  [0.04; 0.62] |
|  | BV ceased | 0.52  [0.19; 1.45] | 0.48  [0.12; 2.02] | 0.4  [0.13; 1.21] | 0.37  [0.13; 1.06] | 0.35  [0.12; 1.05] | 0.34  [0.13; 0.89] | 0.34  [0.14; 0.84] | 0.32  [0.1; 0.97] | 0.31  [0.1; 1] | 0.31  [0.11; 0.87] | 0.17  [0.04; 0.73] |
|  | BV + CCNU | 0.6  [0.37; 0.98] | 0.56  [0.2; 1.55] | 0.46  [0.2; 1.08] | 0.43  [0.26; 0.7] | 0.41  [0.23; 0.72] | 0.4  [0.31; 0.5] | 0.39  [0.23; 0.67] | 0.36  [0.2; 0.66] | 0.36  [0.18; 0.71] | 0.36  [0.24; 0.56] | 0.2  [0.05; 0.72] |
|  | HSPPC-96 + concomitant BV | 0.59  [0.22; 1.54] | 0.54  [0.14; 2.09] | 0.45  [0.21; 0.95] | 0.42  [0.16; 1.12] | 0.4  [0.15; 1.11] | 0.39  [0.16; 0.94] | 0.38  [0.17; 0.86] | 0.36  [0.12; 1] | 0.35  [0.12; 1.03] | 0.35  [0.14; 0.9] | 0.19  [0.05; 0.82] |
|  | FTM | 0.61  [0.25; 1.49] | 0.57  [0.15; 2.02] | 0.47  [0.18; 1.23] | 0.44  [0.18; 1.07] | 0.42  [0.16; 1.04] | 0.4  [0.18; 0.87] | 0.4  [0.2; 0.79] | 0.37  [0.14; 0.97] | 0.37  [0.14; 1.01] | 0.37  [0.16; 0.86] | 0.2  [0.05; 0.8] |
|  | BV + CBP | 0.65  [0.29; 1.47] | 0.6  [0.18; 2.02] | 0.5  [0.21; 1.22] | 0.47  [0.21; 1.05] | 0.44  [0.19; 1.03] | 0.43  [0.22; 0.86] | 0.42  [0.23; 0.77] | 0.39  [0.16; 0.95] | 0.39  [0.15; 0.99] | 0.39  [0.18; 0.87] | 0.21  [0.05; 0.82] |
|  | BV | 0.65  [0.35; 1.21] | 0.6  [0.2; 1.79] | 0.5  [0.24; 1.03] | 0.47  [0.25; 0.86] | 0.44  [0.22; 0.87] | 0.43  [0.28; 0.67] | 0.42  [0.31; 0.58] | 0.39  [0.19; 0.81] | 0.39  [0.18; 0.85] | 0.39  [0.22; 0.7] | 0.21  [0.06; 0.73] |
|  | BV + DST | 0.67  [0.29; 1.56] | 0.63  [0.18; 2.2] | 0.52  [0.21; 1.32] | 0.48  [0.21; 1.12] | 0.46  [0.19; 1.11] | 0.44  [0.22; 0.94] | 0.44  [0.23; 0.85] | 0.41  [0.17; 1.03] | 0.4  [0.16; 1.05] | 0.41  [0.18; 0.93] | 0.22  [0.05; 0.85] |
|  | BV + VRS | 0.74  [0.3; 1.8] | 0.69  [0.19; 2.44] | 0.57  [0.22; 1.52] | 0.53  [0.22; 1.31] | 0.5  [0.19; 1.29] | 0.49  [0.23; 1.06] | 0.48  [0.24; 0.98] | 0.45  [0.17; 1.18] | 0.45  [0.16; 1.22] | 0.45  [0.19; 1.08] | 0.24  [0.06; 0.97] |
|  | CCNU + CDR |  | 0.93  [0.34; 2.44] | 0.77  [0.29; 2.03] | 0.72  [0.51; 1.03] | 0.68  [0.35; 1.33] | 0.66  [0.43; 1.03] | 0.65  [0.33; 1.32] | 0.61  [0.29; 1.25] | 0.6  [0.27; 1.3] | 0.6  [0.34; 1.07] | 0.33  [0.08; 1.28] |
|  | CDR + GFT |  |  | 0.82  [0.23; 3.15] | 0.77  [0.32; 1.93] | 0.73  [0.24; 2.31] | 0.71  [0.26; 1.96] | 0.7  [0.23; 2.27] | 0.65  [0.21; 2.04] | 0.65  [0.2; 2.09] | 0.65  [0.23; 1.92] | 0.35  [0.07; 1.88] |
|  | HSPPC-96 + BV at progression |  |  |  | 0.94  [0.36; 2.41] | 0.89  [0.33; 2.46] | 0.86  [0.37; 2.05] | 0.85  [0.38; 1.89] | 0.79  [0.28; 2.18] | 0.78  [0.27; 2.29] | 0.79  [0.31; 2.03] | 0.43  [0.1; 1.85] |
|  | CDR |  |  |  |  | 0.94  [0.49; 1.88] | 0.92  [0.6; 1.43] | 0.91  [0.46; 1.8] | 0.84  [0.41; 1.7] | 0.83  [0.38; 1.8] | 0.84  [0.47; 1.5] | 0.46  [0.11; 1.8] |
|  | Rego |  |  |  |  |  | 0.97  [0.58; 1.63] | 0.96  [0.45; 2.03] | 0.9  [0.41; 1.9] | 0.89  [0.39; 2.02] | 0.89  [0.47; 1.65] | 0.49  [0.12; 2.02] |
|  | CCNU |  |  |  |  |  |  | 0.99  [0.57; 1.7] | 0.92  [0.52; 1.6] | 0.91  [0.47; 1.72] | 0.92  [0.63; 1.32] | 0.5  [0.13; 1.85] |
|  | NIVO |  |  |  |  |  |  |  | 0.93  [0.42; 2.01] | 0.92  [0.4; 2.09] | 0.93  [0.48; 1.77] | 0.5  [0.14; 1.75] |
|  | CCNU + GAL |  |  |  |  |  |  |  |  | 0.99  [0.58; 1.74] | 1  [0.5; 1.95] | 0.54  [0.13; 2.25] |
|  | GAL |  |  |  |  |  |  |  |  |  | 1.01  [0.48; 2.12] | 0.55  [0.12; 2.38] |
|  | ENZA |  |  |  |  |  |  |  |  |  |  | 0.55  [0.14; 2.12] |
